# Nonlinear Stability Analysis of Eccentrically Stiffened Functionally Graded Truncated Conical Sandwich Shells with Porosity

**DOI:** 10.3390/ma11112200

**Published:** 2018-11-06

**Authors:** Duc-Kien Thai, Tran Minh Tu, Le Kha Hoa, Dang Xuan Hung, Nguyen Ngọc Linh

**Affiliations:** 1 Department of Civil and Environmental Engineering, Sejong University, 98 Gunja-dong, Gwangjin-gu, Seoul 143-747, Korea; thaiduckien@sejong.ac.kr; 2 Faculty of Industrial and Civil Engineering, University of Civil Engineering, Hanoi 100000, Vietnam; dangxuanhung@gmail.com (D.X.H.); nguyenngoclinh@gmail.com (N.N.L.); 3 Division of Computational Mathematics and Engineering, Institute for Computational Science, Ton Duc Thang University, Ho Chi Minh City 700000, Vietnam; lekhahoa@tdtu.edu.vn; 4 Faculty of Civil Engineering, Ton Duc Thang University, Ho Chi Minh City 700000, Vietnam

**Keywords:** porous materials, truncated conical sandwich shell, metal foam core layer, non-linear buckling analysis, orthogonal stiffener, elastic foundation

## Abstract

This paper analyzes the nonlinear buckling and post-buckling characteristics of the porous eccentrically stiffened functionally graded sandwich truncated conical shells resting on the Pasternak elastic foundation subjected to axial compressive loads. The core layer is made of a porous material (metal foam) characterized by a porosity coefficient which influences the physical properties of the shells in the form of a harmonic function in the shell’s thickness direction. The physical properties of the functionally graded (FG) coatings and stiffeners depend on the volume fractions of the constituents which play the role of the exponent in the exponential function of the thickness direction coordinate axis. The classical shell theory and the smeared stiffeners technique are applied to derive the governing equations taking the von Kármán geometrical nonlinearity into account. Based on the displacement approach, the explicit expressions of the critical buckling load and the post-buckling load-deflection curves for the sandwich truncated conical shells with simply supported edge conditions are obtained by applying the Galerkin method. The effects of material properties, core layer thickness, number of stiffeners, dimensional parameters, semi vertex angle and elastic foundation on buckling and post-buckling behaviors of the shell are investigated. The obtained results are validated by comparing with those in the literature.

## 1. Introduction

Functionally graded (FG) materials are microscopically nonhomogeneous materials with smoothly and continuously varying mechanical properties in the preferred directions. The advantages of functionally graded material (FGM) include avoiding crack, avoiding delamination and eliminating residual stress. In micromechanics, FGM is considered to contain porosity during the production process, these porosities could be characterized to obtain the expected material properties such as the local density and to obtain the expected structural performance. Furthermore, porous materials such as metal foams have excellent energy-absorbing capability forming an important category of lightweight materials. As a result, porous materials have been considered in a wide range of application in practice for structures subjected to dynamic or impact loadings.

Truncated conical shells have been utilized in various engineering activities such as aerospace engineering, marine and ocean engineering structures, components of missiles and spacecrafts and nuclear reactors. Metallic sandwich structures are widely used in the aviation industry as well as in ship and railway engineering because of their low density, high specific strength, and effective energy absorption. The buckling and post-buckling behaviors of FG shells in cylindrical and conical forms under mechanical and thermal loads are prominent topics, drawing the considerable attention of many researchers. Huang and Han [1] used Donell shell theory to study the stability characteristics of functionally graded shells in cylindrical forms subjected to axially compressive loads employing the Ritz energy method. Naj et al. [2] analyze the instability of FG truncated conical shells under the coupling of thermal and mechanical loadings using the first-order shell theory. Sofiyev and his colleagues [3,4,5,6,7,8,9,10] published many studies on linear and nonlinear buckling of FG cylindrical and conical shells. By applying the Galerkin method and smeared stiffeners technique, Duc and his colleagues [11,12,13,14,15,16,17] investigated buckling and post-buckling behaviors of FG cylindrical and conical shells reinforced by eccentrically stiffeners (ES). Using the same approach, Bich et al. [18,19,20] examined the buckling behaviors and dynamic stability characteristics of eccentrically stiffened FG cylindrical shells and panels. Recently, Dung et al. [21,22] presented the theoretical solution for the buckling behaviors of FG truncated conical shells under different of mechanical loadings such as uniformly distributed loads and axially compressive loads. Dung and Chan [23] analyzed the orthogonally stiffened FG truncated conical shells in terms of the mechanical stability. Dung et al. [24] analyzed the nonlinear post-buckling behaviors of the eccentrically orthogonal stiffened FG truncated conical shells.

There are a few studies on the buckling of FG porous plates and beams in the available literature. Magnucki and Stasiewicz [25] examined the buckling features of beams with porosity considering the total potential energy using elastic formulations. Magnucka-Blandzi [26,27] mathematically modeled a porous sandwich plate to determine critical in-plane compressed loads. The work of Magnucka-Blandzi [28] focused on axis-symmetrical deflection and buckling of simply supported circular porous–cellular plates under lateral uniformly distributed pressures and compressive pressures in the radial direction uniform. Static buckling and bending features of FG beams with porosity taking the shear deformation into account are studied by Chen et al. in [29]. Kitipornchai et al. [30] studied elastic buckling and free vibration behaviors of closed-cell beams made of metal foam and reinforced by graphene platelets. Jabbari et al. [31] examined the buckling behaviors of an FG thin circle-shaped plate made of saturated porous materials. In another study, he also examined the buckling behaviors of a porous circular plate subjected to radial loadings employing the higher-order shear deformation theory [32]. To control the formation of porous structures, fabrication parameters need to be managed. In microelectromechanical systems (MEMS) and nanoelectromechanical systems (NEMS), we can improve the physical characteristic of micro/nano-scale structures by tailoring the architecture of porous materials. Examination and assessment of size-effects in NEMs structural problems, many researchers have been focused on size-dependent mechanical models [33,34,35,36]. Size effect plays important role in micron and sub-micron scales of metallic materials. Size effects in elastic-plastic functionally graded materials (FGMs) have been reported in work of Mathew et al. [37], Martínez-Paneda et al. [38,39].

From the above-mentioned literature context, it can be seen that there are very few studies focused on linear and non-linear stability of eccentrically stiffened FGM truncated conical shells. To the best of our knowledge, there are no publications on the nonlinear stability behaviors of the eccentrically stiffened functionally graded truncated conical sandwich shells with the porous core layer. The aim of the present paper is to meet this demand. The porous material core layer of the shell is made of metal foam. The outer and inner layers, eccentrically orthogonal stiffener systems are made of FGM. The shell is supported by Pasternak elastic foundation and subjected to the axial compressive load. The classical shell theory, the smeared stiffener technique, and the Galerkin method are applied to come up with explicit expressions of the critical buckling load and the post-buckling load-deflection curves for sandwich truncated conical shells with simply supported edge conditions. The effects of material properties, the number of stiffeners, geometry parameters, and elastic foundation on stability behaviors of the shell are also examined.

## 2. Model Configurations and Elastic Foundations

**Figure 1:**
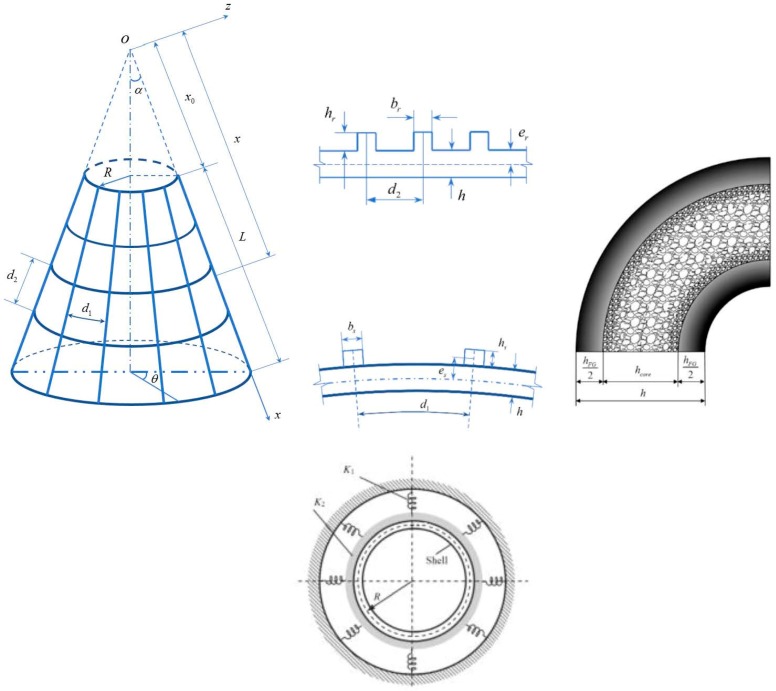
Geometry configurations and coordinates of the PSTC.

A porous eccentrically stiffened functionally graded truncated conical sandwich shells (PSTC) is considered with the geometry configurations and the coordinate system being shown in Figure 1. In which, α denotes the semi-vertex angle, R denotes the small base radius of the shell, L denotes the slant height and h denotes the shell thickness.

The shell consists of inner and outer layers (layers 1 and 3) made of FGM of the thickness $h_{FG}$, and the porous core layer (layer 2) of the thickness $h_{core}$. The PSTC is located in a curvilinear coordinate (x,θ,z) in which x and z axis share the origin at the vertex of the conical shell and together form a plane through the symmetry line of the shell. x axis exists along the shell slant and z axis is at right angles to the slant line. It is noted that the origin is located in the mid-surface of the shell and $x_{0}$ denotes the virtual slant height from the vertex to the adjacent base of the shell. Corresponding to x,θ and z axes, there are three displacements components u,v, and w of a point in the mid-surface, respectively. The displacement along the z axis (w) is also called the deflection of the PSTC which is also the primary variable of this work.

The space between FG stiffeners is assumed to be constant and closely spaced in the outer face of the PSTC. The Young moduli of FG cover layers and stiffeners vary according to a simple power distribution through the z direction with the exponent is the volume fraction of the constituents, and the Young moduli of the core follow a simple cosine rule of a symmetric distribution defined as follows:

(1a) $$E_{sh}=\left\{ \begin{array}{ll} E_{c}+E_{mc}{\left(\frac{2z+h_{FG}+h_{core}}{h_{FG}}\right)}^{k} & \mathrm{at}\;-\frac{h}{2}\leq z\leq -\frac{h_{core}}{2}\\ E_{m}\left[1-e_{0}\cos\left(\frac{\pi z}{h_{core}}\right)\right] & \mathrm{at}\;-\frac{h_{core}}{2}\leq z\leq \frac{h_{core}}{2}\\ E_{c}+E_{mc}{\left(\frac{-2z+h_{FG}+h_{core}}{h_{FG}}\right)}^{k} & \mathrm{at}\;\frac{h_{core}}{2}\leq z\leq \frac{h}{2} \end{array}\right.\;$$

(1b) $$\{\begin{array}{c} h=h_{core}+h_{FG}\\ 0<e_{0}<1 \end{array}\;$$

Reinforced stiffeners are considered in two following cases.

**Case 1:** Inside FGM stiffener

(2a) $$\begin{array}{ll} E_{s}=E_{c}+E_{mc}{\left(\frac{2z-h}{2h_{s}}\right)}^{k_{2}} & \mathrm{at}\;\frac{h}{2}\leq z\leq \frac{h}{2}+h_{s}\\ E_{r}=E_{c}+E_{mc}{\left(\frac{2z-h}{2h_{r}}\right)}^{k_{3}} & \mathrm{at}\;\frac{h}{2}\leq z\leq \frac{h}{2}+h_{r} \end{array}$$

**Case 2:** Outside FGM stiffener

(2b) $$\begin{array}{ll} E_{s}=E_{c}+E_{mc}{\left(-\frac{2z+h}{2h_{s}}\right)}^{k_{2}} & \mathrm{at}\;-\frac{h}{2}-h_{s}\leq z\leq -\frac{h}{2}\\ E_{r}=E_{c}+E_{mc}{\left(-\frac{2z+h}{2h_{r}}\right)}^{k_{3}} & \mathrm{at}\;-\frac{h}{2}-h_{r}\leq z\leq -\frac{h}{2} \end{array}$$

where:

$h_{FG}/2$ is the FG coating thickness,

$E_{mc}=E_{m}-E_{c},\;E_{cm}=E_{c}-E_{m}$,
$h_{core}$ is the core layer thickness,
$h_{s}$, $h_{r}$ denote stringers and rings thickness respectively,
$e_{0}$ is the porosity coefficient of the core layer,
*k*, *k_2_*, and *k_3_* are the shell, stringers, and rings volume fraction indexes respectively.
sh,m,c,r, and s denote shell, metal, ceramic, ring, and stringer respectively.
st denotes stiffeners in general, stiffeners are stringers and rings.
$E_{c},E_{m}$ are Young’s moduli of ceramic and metal.
$E_{sh},E_{s},$ and $E_{r}$ are the Young moduli of shell, stringer, and ring of materials respectively.

The Poisson’s ratios v of the shell and stiffeners materials are assumed to be independent of thickness coordinate [6].

It is noted from Equations (1) and (2) that the continuous variations of the material properties are satisfied between layers of the PSTC. From Equation (1), we can obtain equations for these different cases, namely the FG sigmoid sandwich shell with $\left(h_{core}=0\right)$, the metal foam sandwich shell with FG face sheets $\left(e_{0}=0\right)$, or the full metal shell $\left(e_{0}=k=0\right)$.

The reaction of the elastic foundation on the conical shell is described by using the Pasternak model. The shell-foundation interaction may be expressed as [40]

(3) $$q_{f}=K_{1}w-K_{2}\left(\frac{\partial^{2}w}{\partial x^{2}}+\frac{1}{x}\frac{\partial w}{\partial x}+\frac{1}{x^{2}\sin^{2}\alpha }\frac{\partial^{2}w}{\partial \theta^{2}}\right)\;$$

where $K_{1}$ (N/m^3^) and $K_{2}$ (N/m) respectively are the Winkler foundation stiffness and the shear subgrade modulus of the foundation.

## 3. Theoretical Formulations

From the Donnell shell theory, at a distance z from the mid-surface of the shell, the normal and shear strains are given as follows [41]:

(4) $$\varepsilon_{x}=\varepsilon_{xm}+zk_{x},\;\varepsilon_{\theta }=\varepsilon_{\theta m}+zk_{\theta },\;\gamma_{x\theta }=\gamma_{x\theta m}+2zk_{x\theta }\;$$

in which $\varepsilon_{xm}$ and $\varepsilon_{\theta m}$ are the normal strains $\gamma_{x\theta m}$ is the shear strain at a point on the shell mid-surface, and $k_{x},k_{\theta },k_{x\theta }$ are bending and twisting curvatures with respect to the x-axis, θ-axis, and the plane (x,θ), respectively. Considering the von Karman geometrical nonlinearity, the strain–displacement relations are defined as [41]

(5) $$\begin{array}{l} \varepsilon_{xm}=\frac{\partial u}{\partial x}+\frac{1}{2}{\left(\frac{\partial w}{\partial x}\right)}^{2},\;\varepsilon_{\theta m}=\frac{1}{x\sin\alpha }\frac{\partial v}{\partial \theta }+\frac{u}{x}+\frac{w}{x}\cot\alpha +\frac{1}{2x^{2}\sin^{2}\alpha }{\left(\frac{\partial w}{\partial \theta }\right)}^{2},\\ \gamma_{x\theta m}=\frac{1}{x\sin\alpha }\frac{\partial u}{\partial \theta }-\frac{v}{x}+\frac{\partial v}{\partial x}+\frac{1}{x\sin\alpha }\frac{\partial w}{\partial x}\frac{\partial w}{\partial \theta },\\ k_{x}=-\frac{\partial^{2}w}{\partial x^{2}},\;k_{\theta }=-\frac{1}{x^{2}\sin^{2}\alpha }\frac{\partial^{2}w}{\partial \theta^{2}}-\frac{1}{x}\frac{\partial w}{\partial x},\;k_{x\theta }=-\frac{1}{x\sin\alpha }\frac{\partial^{2}w}{\partial x\partial \theta }+\frac{1}{x^{2}\sin\alpha }\frac{\partial w}{\partial \theta } \end{array}$$

The generalized Hooke law for the conical shell is presented as follows:

(6) $$\sigma_{x}^{sh}=\frac{E\left(z\right)}{1-\nu^{2}}\left(\varepsilon_{x}+\nu \varepsilon_{\theta }\right),\;\sigma_{\theta }^{sh}=\frac{E\left(z\right)}{1-\nu^{2}}\left(\varepsilon_{\theta }+\nu \varepsilon_{x}\right),\;\sigma_{x\theta }^{sh}=\frac{E\left(z\right)}{2\left(1+\nu \right)}\gamma_{x\theta }\;$$

and for the stringer and ring stiffeners,

(7) $$\sigma_{x}^{st}=E_{s}\varepsilon_{x},\;\sigma_{\theta }^{st}=E_{r}\varepsilon_{\theta }\;$$

The material of the stiffeners is similar to the material of the FG coating at the outer surface. If the outside surface of the FG coating is ceramic-rich, the material of the stiffeners is ceramic, and vice versa.

Considering the change of stringers spacing, applying the Lekhnitskii smeared stiffener technique, and omitting the twisting effects of the stiffeners, we can define the force and moment resultants of the PSTC as follows:

(8) $$\begin{array}{lll} N_{x}=\int_{-h/2}^{h/2}\sigma_{x}^{sh}dz+\frac{b_{s}}{d_{1}\left(x\right)}\int_{h/2}^{h/2+h_{s}}\sigma_{x}^{s}dz, & N_{\theta }=\int_{-h/2}^{h/2}\sigma_{\theta }^{sh}dz+\frac{b_{r}}{d_{2}}\int_{h/2}^{h/2+h_{r}}\sigma_{\theta }^{s}dz, & N_{x\theta }=\int_{-h/2}^{h/2}\sigma_{x\theta }dz\;\\ M_{x}=\int_{-h/2}^{h/2}z\sigma_{x}^{sh}dz+\frac{b_{s}}{d_{1}\left(x\right)}\int_{h/2}^{h/2+h_{s}}z\sigma_{x}^{s}dz, & M_{\theta }=\int_{-h/2}^{h/2}z\sigma_{\theta }^{sh}dz+\frac{b_{r}}{d_{2}}\int_{h/2}^{h/2+h_{r}}z\sigma_{\theta }^{s}dz, & M_{x\theta }=\int_{-h/2}^{h/2}z\sigma_{x\theta }dz \end{array}\;$$

Introducing Equations (6) and (7) into Equation (8) we obtain [22]

(9) $$\begin{array}{l} \left\{\begin{array}{c} N_{x}\\ N_{\theta }\\ N_{x\theta } \end{array}\right\}=\left[\begin{array}{ccc} A_{11}+\frac{E_{1s}b_{s}}{d_{1}\left(x\right)} & A_{12} & 0\\ A_{12} & A_{22}+\frac{E_{1r}b_{r}}{d_{2}} & 0\\ 0 & 0 & A_{66} \end{array}\right]\left\{\begin{array}{c} \varepsilon_{xm}\\ \varepsilon_{\theta m}\\ \gamma_{x\theta m} \end{array}\right\}+\left[\begin{array}{ccc} B_{11}+C_{1}\left(x\right) & B_{12} & 0\\ B_{12} & B_{22}+C_{2} & 0\\ 0 & 0 & 2B_{66} \end{array}\right]\left\{\begin{array}{c} k_{x}\\ k_{\theta }\\ k_{x\theta } \end{array}\right\}\;\\ \left\{\begin{array}{c} M_{x}\\ M_{\theta }\\ M_{x\theta } \end{array}\right\}=\left[\begin{array}{ccc} B_{11}+C(x) & B_{12} & 0\\ B_{12} & B_{22}+C_{2} & 0\\ 0 & 0 & B_{66} \end{array}\right]\left\{\begin{array}{c} \varepsilon_{xm}\\ \varepsilon_{\theta m}\\ \gamma_{x\theta m} \end{array}\right\}+\left[\begin{array}{ccc} D_{11}+\frac{E_{3s}b_{s}}{d_{1}\left(x\right)} & D_{12} & 0\\ D_{12} & D_{22}+\frac{E_{3r}b_{r}}{d_{2}} & 0\\ 0 & 0 & 2D_{66} \end{array}\right]\left\{\begin{array}{c} k_{x}\\ k_{\theta }\\ k_{x\theta } \end{array}\right\}\; \end{array}$$

in which the coefficients are presented in Appendix A.

The nonlinear equations of equilibrium of the PSTC resting on Pasternak foundation using the Donnell shell theory are given as follows [22]:

(10) $$\begin{array}{l} xN_{x,x}+\frac{1}{\sin\alpha }N_{x\theta ,\theta }+N_{x}-N_{\theta }=0\\ \frac{1}{\sin\alpha }N_{\theta ,\theta }+xN_{x\theta ,x}+2N_{x\theta }=0\\ xM_{x,xx}+2M_{x,x}+\frac{2}{\sin\alpha }\left(M_{x\theta ,x\theta }+\frac{1}{x}M_{x\theta ,\theta }\right)+\frac{1}{x\sin^{2}\alpha }M_{\theta ,\theta \theta }-M_{\theta ,x}-N_{\theta }\cot\alpha \\ \;+{\left(xN_{x}w_{,x}+\frac{1}{\sin\alpha }N_{x\theta }w_{,\theta }\right)}_{,x}+\frac{1}{\sin\alpha }{\left(N_{x\theta }w_{,x}+\frac{1}{x\sin\alpha }N_{\theta }w_{,\theta }\right)}_{,\theta }+{\left(xN_{x}^{o}w_{,x}\right)}_{,x}\\ -xK_{1}w+xK_{2}\left(\frac{\partial^{2}w}{\partial x^{2}}+\frac{1}{x}\frac{\partial w}{\partial x}+\frac{1}{x^{2}\sin^{2}\alpha }\frac{\partial^{2}w}{\partial \theta^{2}}\right)=0\; \end{array}$$

where x,z and θ following the comma symbol (,) indicates the partial derivative with respect to x,z and θ, respectively.

## 4. Prebuckling State Analysis

In this section, the PSTC is considered solely exposed to an axial compression P at the small base $x=x_{0}$. The equilibrium equations of the PSTC in the membrane-like form is derived from Equation (10) taking the symmetry of geometry and loading characteristics into account as follows:

(11) $$x\frac{dN_{x}^{0}}{dx}+N_{x}^{0}-N_{\theta }^{0}=0,\;N_{x\theta }^{0}=0,\;-N_{\theta }^{0}\cot\alpha =0\;$$

Solving this system with condition

(12) $$N_{x}^{0}=-\frac{P}{\cos\alpha }\;$$

We obtain the prebuckling force resultants

(13) $$N_{x}^{o}=-\frac{px_{o}}{x\cos\alpha },\;N_{\theta }^{0}=0,\;N_{x\theta }^{0}=0\;$$

or in another form

(14) $$N_{x}^{o}=-\frac{P}{\pi x\sin2\alpha },\;\mathrm{where}\;P=2\pi px_{o}\sin\alpha \;$$

## 5. Nonlinear Stability Formulations

Introducing Equation (4) into Equation (9) we obtain the force and moment resultants in term of displacements. The results are then substituted into Equation (10) in conjunction with Equation (14), and we have the stability equations as follows:

(15) $${ℜ}_{11}\left(u\right)+{ℜ}_{12}\left(v\right)+{ℜ}_{13}\left(w\right)+G_{14}=0\;$$

(16) $${ℜ}_{21}\left(u\right)+{ℜ}_{22}\left(v\right)+{ℜ}_{23}\left(w\right)+G_{24}=0\;$$

(17) $${ℜ}_{31}\left(u\right)+{ℜ}_{32}\left(v\right)+{ℜ}_{33}\left(w\right)+P{ℜ}_{34}\left(w\right)+G_{34}=0\;$$

where ${ℜ}_{ij}$ with i=(1−3) and j=(1−4) are linear differential operators and $G_{ij}$ with i=(1−3) and j=4 are nonlinear components, these values are listed in Appendix B. Equations (15)–(17) are employed to compute the critical buckling load and analyze post-buckling behavior of the PSTC. However, these equations are the coupling nonlinear partial differential equations whose difficulty would be overcome in the following section.

## 6. Buckling and Post-Buckling Analysis

The PSTC is considered simply supported at two bases such that

(18) $$v=w=0,\;M_{x}=0\;at\;x=x_{o},x_{o}+L\;$$

The solution approximately satisfying Equation (18) are chosen as [22,24]

(19) $$\begin{array}{c} u=U\cos\frac{m\pi (x-x_{0})}{L}\sin\frac{n\theta }{2}\\ v=V\sin\frac{m\pi (x-x_{0})}{L}\cos\frac{n\theta }{2}\\ w=W\sin\frac{m\pi (x-x_{0})}{L}\sin\frac{n\theta }{2}\; \end{array}$$

where n is the quantity of full-waves in the circumferential direction of the shell, and m is the number of half-waves along x axis. *U*, *V* and W are the corresponding displacement amplitudes which would be determined by then. In the integration domain given as $x_{0}\leq x\leq x_{0}+L$ and 0≤θ≤2π, Equations (15) and (16) are weighted by x and Equation (17) is weighted by $x^{2}$ before employing the Galerkin method to the obtained results. We have

(20) $$\begin{array}{c} J_{1}=\int_{x_{o}}^{x_{o}+L}\int_{0}^{2\pi }\Omega_{1}\sin\frac{n\theta }{2}\cos\frac{m\pi (x-x_{0})}{L}\sin\alpha d\theta dx\\ J_{2}=\int_{x_{o}}^{x_{o}+L}\int_{0}^{2\pi }\Omega_{2}\cos\frac{n\theta }{2}\sin\frac{m\pi (x-x_{0})}{L}\sin\alpha d\theta dx\\ J_{3}=\int_{x_{o}}^{x_{o}+L}\int_{0}^{2\pi }\Omega_{3}\sin\frac{n\theta }{2}\sin\frac{m\pi (x-x_{0})}{L}\sin\alpha d\theta dx\; \end{array}$$

where

(21) $$\begin{array}{c} \Omega_{1}=x\left[{ℜ}_{11}\left(u\right)+{ℜ}_{12}\left(v\right)+{ℜ}_{13}\left(w\right)+G_{14}\right]\\ \Omega_{2}=x\left[{ℜ}_{21}\left(u\right)+{ℜ}_{22}\left(v\right)+{ℜ}_{23}\left(w\right)+G_{24}\right]\\ \Omega_{3}=x^{2}\left[{ℜ}_{31}\left(u\right)+{ℜ}_{32}\left(v\right)+{ℜ}_{33}\left(w\right)+P{ℜ}_{34}\left(w\right)+G_{34}\right]\; \end{array}$$

Introducing Equation (19) into Equation (21) and then the results into Equation (20), after integrations and other manipulations, we obtain

(22) $$H_{11}U+H_{12}V+H_{13}W+L_{14}W^{2}=0\;$$

(23) $$H_{21}U+H_{22}V+H_{23}W+L_{24}W^{2}=0\;$$

(24) $$H_{31}U\;+H_{32}V+\left(H_{33}+H_{34}P\right)W+L_{34}W^{2}+L_{35}VW+L_{36}UW+L_{37}W^{3}=0\;$$

where $H_{ij}$ and $L_{ij}$ are given in Appendix C.

We obtain the expression for *U* and *V* from Equations (22) and (23) as follows:

$$\begin{array}{c} U=\frac{H_{13}H_{22}-H_{12}H_{23}}{H_{12}H_{21}-H_{11}H_{22}}W+\frac{L_{14}H_{22}-L_{24}H_{12}}{H_{12}H_{21}-H_{11}H_{22}}W^{2}\\ V=\frac{H_{11}H_{23}-H_{13}H_{21}}{H_{12}H_{21}-H_{11}H_{22}}W+\frac{L_{24}H_{11}-L_{14}H_{21}}{H_{12}H_{21}-H_{11}H_{22}}W^{2}\; \end{array}$$

Substituting *U* and *V* into Equation (24) we obtain the following equation.

(25) $$\begin{array}{c} \left(\frac{L_{35}L_{24}H_{11}-L_{35}L_{14}H_{21}-L_{36}L_{24}H_{12}+L_{36}L_{14}H_{22}}{H_{12}H_{21}-H_{11}H_{22}}+L_{37}\right)W^{3}\\ +\left(\begin{array}{l} \frac{-H_{31}L_{24}H_{12}+H_{31}L_{14}H_{22}+H_{32}L_{24}H_{11}-H_{32}L_{14}H_{21}}{H_{12}H_{21}-H_{11}H_{22}}+L_{34}\\ \frac{-L_{35}H_{13}H_{21}+L_{35}H_{11}H_{23}-L_{36}H_{12}H_{23}+L_{36}H_{13}H_{22}}{H_{12}H_{21}-H_{11}H_{22}} \end{array}\right)W^{2}\\ +\left(\frac{H_{31}H_{13}H_{22}-H_{31}H_{12}H_{23}-H_{32}H_{13}H_{21}+H_{32}H_{11}H_{23}}{H_{12}H_{21}-H_{11}H_{22}}+H_{33}\right)W+H_{34}PW=0\; \end{array}$$

Solving the Equation (25), the analytical expression of *P* is obtained as follows:

(26) $$\begin{array}{l} P=\frac{1}{H_{34}}\left(\frac{L_{35}L_{14}H_{21}-L_{35}L_{24}H_{11}+L_{36}L_{24}H_{12}-L_{36}L_{14}H_{22}}{H_{12}H_{21}-H_{11}H_{22}}-L_{37}\right)W^{2}\\ \;+\frac{1}{H_{34}}\left(\begin{array}{l} \frac{H_{31}L_{24}H_{12}-H_{31}L_{14}H_{22}+H_{32}L_{14}H_{21}-H_{32}L_{24}H_{11}}{H_{12}H_{21}-H_{11}H_{22}}-L_{34}\\ +\frac{L_{35}H_{13}H_{21}-L_{35}H_{11}H_{23}+L_{36}H_{12}H_{23}-L_{36}H_{13}H_{22}}{H_{12}H_{21}-H_{11}H_{22}} \end{array}\right)W\\ +\frac{1}{H_{34}}\left(\frac{H_{31}H_{12}H_{23}-H_{31}H_{13}H_{22}+H_{32}H_{13}H_{21}-H_{32}H_{11}H_{23}}{H_{12}H_{21}-H_{11}H_{22}}-H_{33}\right)\; \end{array}$$

By then, the critical buckling load and the post-buckling load-deflection curve of the PSTC subjected to axial compressive loads could be obtained from Equation (26).

Setting W→0, Equation (26) yields the upper buckling compressive load as follows:

(27) $$P=P_{upper}=\frac{1}{H_{34}}\left(\frac{H_{31}H_{12}H_{23}-H_{31}H_{13}H_{22}+H_{32}H_{13}H_{21}-H_{32}H_{11}H_{23}}{H_{12}H_{21}-H_{11}H_{22}}-H_{33}\right)\;$$

It is clear from Equation (26) and (27) that, the value of the buckling loads depends on m and n, as a result, it is worth considering the values of m and n in making these loads reaches the minimum values.

## 7. Numerical Results and Discussion

The geometric parameters of various model of truncated conical shell and stiffeners used in the present study are listed in Table 1.

### 7.1. Verification Study

To verify the present study, firstly, the dimensionless buckling axial compressive loads $P^{*}$ of single layer pure isotropic (Stainless steel—SUS304) un-stiffened truncated conical shell by setting $\left(h_{FG}=0,e_{0}=0\right)$ are compared with the results of Naj et al. [2] and Baruch et al. [42]. The results are presented in Table 2, and in this particular case, the circular cylindrical shell of model M1 without elastic foundation is considered. The material properties are ν=0.3, $E_{m}=200$ GPa. We determine $P^{*}=P_{cr}/P_{cl}$ with $P_{cl}=\frac{2\pi Eh^{2}\cos^{2}\alpha }{\sqrt{3\left(1-\nu^{2}\right)}}$ [2] and is found from Equation (27).

The next verification is performed for stiffened FGM sandwich truncated conical shells with metal core $\left(e_{0}=0\right)$, FG faces, and FG stiffeners (Model M2) resting on Pasternak’s foundation. The obtained results are presented in Table 3 and are compared with the linear critical loads $P_{cr}$ of Dung et al. [21]. In which, the Alumina has $E_{c}=380$ GPa, Aluminum has $E_{m}=70\;\mathrm{GPa}$, and ν=0.3 for both constituents. $k_{2}=k_{3}=k=1$, $K_{1}=5\times 10^{5}\;{N/m}^{3}$, and $K_{2}=3\times 10^{4}\;N/m$. The expression $P_{cr}$ is taken from Equation (27).

Finally, Table 4 compares the present results with those of Deniz [43] for un-stiffened three-layered FG/Metal/FG truncated conical shells (Model M3) subjected to an axial load without elastic foundation. The database is used in this example: $E_{c}=348.43$ GPa; $E_{m}=201.04$ GPa; h=0.01 m; α=45°; L/R=2; R/h=150; $K_{1}=K_{2}=0$; $e_{0}=0$. The author analyzed non-linear stability based on the Donnell shell theory with von Karman-type of kinematic non-linearity. Using stress approach and approximated solution with two terms may cause the considerable discrepancy between two results.

From above three verifications, we can conclude that the results of the present study agree well with the existing results in the available literature.

### 7.2. The PSTC on Pasternak Elastic Foundations

In the following subsections, the PSTC resting on Pasternak elastic foundations are considered. FG materials of the coatings are a blend of Si3N4 (Silicon nitride-ceramic) and SUS304 (Stainless steel-metal) with $E_{c}=348.43\;\mathrm{GPa}$ and with $E_{m}=201.04$ GPa and the metal foam of the core layer has $E_{m}=201.04$ GPa. The PSTC’s model is M3 with volume fraction indices $k_{2}=k_{3}=k=1$, and foundation parameters $K_{1}=6\times 10^{7}$ N/m^3^, $K_{2}=4\times 10^{5}$ N/m.

#### 7.2.1. Effect of Porosity Coefficients $e_{0}$ and Thickness of Core Layer $h_{core}$

**Figure 2:**
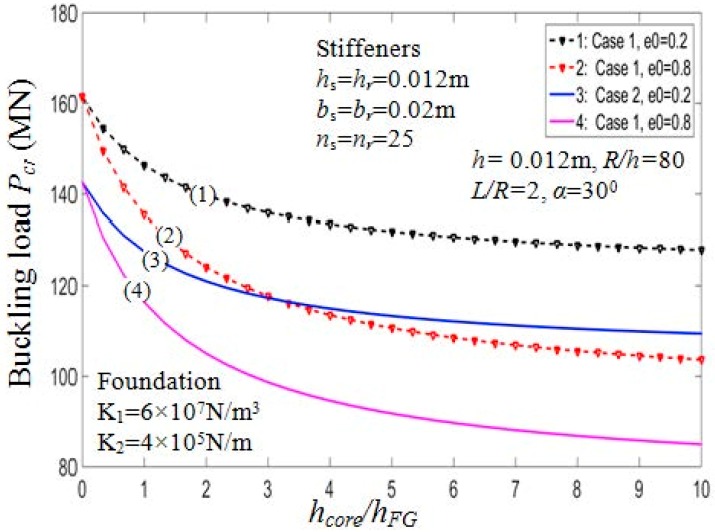
Effects of *h_core_*/*h_FG_* and *e*_0_ on critical load *P_cr_* (*k*_2_ = *k*_3_ = *k* = 1). Case 1: Outside stiffener; Case 2: Inside stiffener.

**Figure 3:**
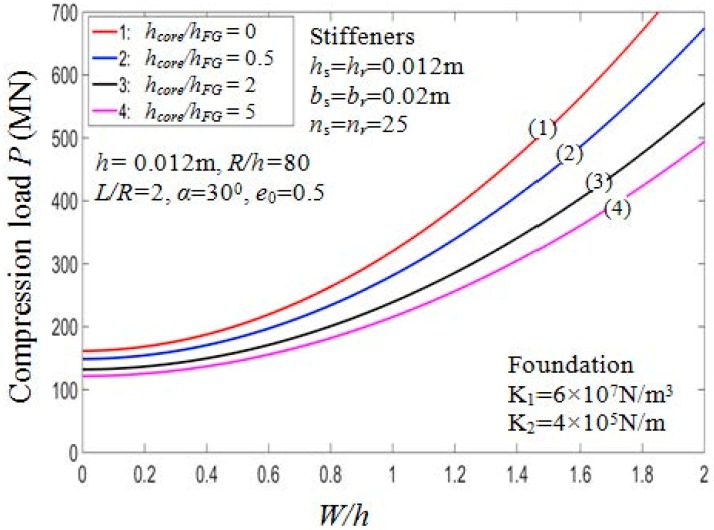
Effects of *h_core_*/*h_FG_* on postbuckling load—deflection curves (Case 1, *k*_2_ = *k*_3_ = *k* = 1).

Table 5 presents the critical buckling loads of the PSTC with different degrees of porosity, $h_{core}/h_{FG}$ ratios, and the buckling mode parameters (*m*,*n*). Furthermore, two cases of stiffeners arrangement, namely outside and inside eccentrically FG stiffeners are considered. Figure 2 and Figure 3 illustrate the ratio $h_{core}/h_{FG}$ effect on the critical buckling loads and post-buckling load-deflection paths of the shell, respectively.

From the figures, it can be seen that when $h_{core}/h_{FG}$ ratios increase, the buckling loads decrease for both cases of arranging stiffeners. Taking case 1, $e_{0}=0.5$ as an example, the critical load decreases by about 43% from $P_{cr}=161.4554$ MN (with $h_{core}/h_{FG}=0$) to $P_{cr}=112.5450$ MN (with $h_{core}/h_{FG}=20$). The stiffener arrangement has considerable influence on the critical buckling loads. Indeed, the $P_{cr}$ value of the PSTC reinforced by inside stiffeners is always smaller than that by outside stiffeners.

**Figure 4:**
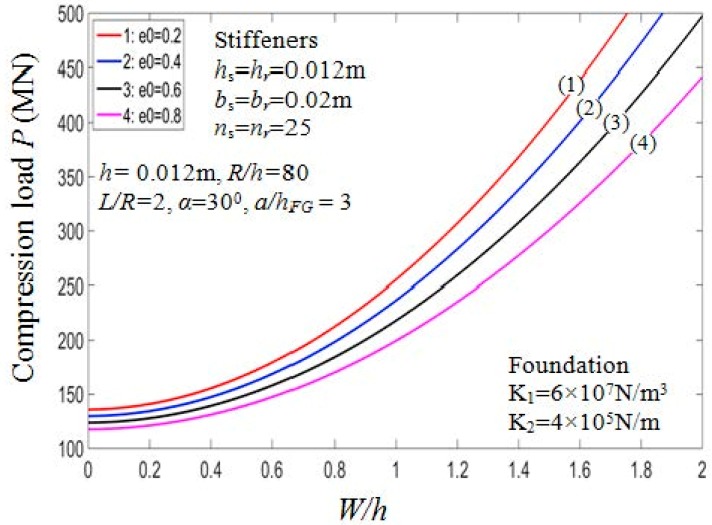
Effects of *e*_0_ on postbuckling load—deflection curves (Outside stiffener, *k*_2_ = *k*_3_ = *k* = 1).

**Figure 5:**
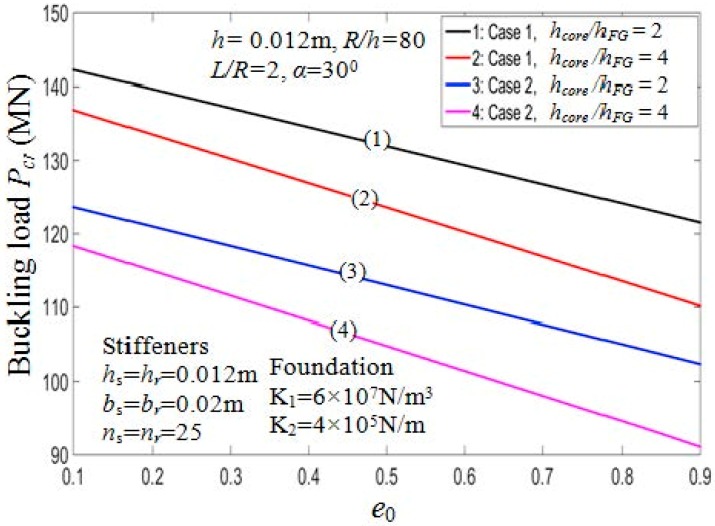
Effects of *e*_0_ on critical load *P_cr_* (*k*_2_ = *k*_3_ = *k* = 1).

Figure 4 depicts the influence of porosity coefficients on the behaviors of the PSTC in the post-buckling phase. From the figure, the loading capacity of the shell decreases when *e*_0_ increases. Figure 5 examines the relation between the critical buckling loads of the PSTC and the porosity coefficients existed in the shell. It is found that with the increment of $e_{0}$, the critical buckling load *P_cr_* of the PSTC decreases. Indeed, the porosity affects the Young modulus of porous shells significantly as can be seen from Equation (1).

#### 7.2.2. Effect of Semi-Vertex Angle α

**Figure 6:**
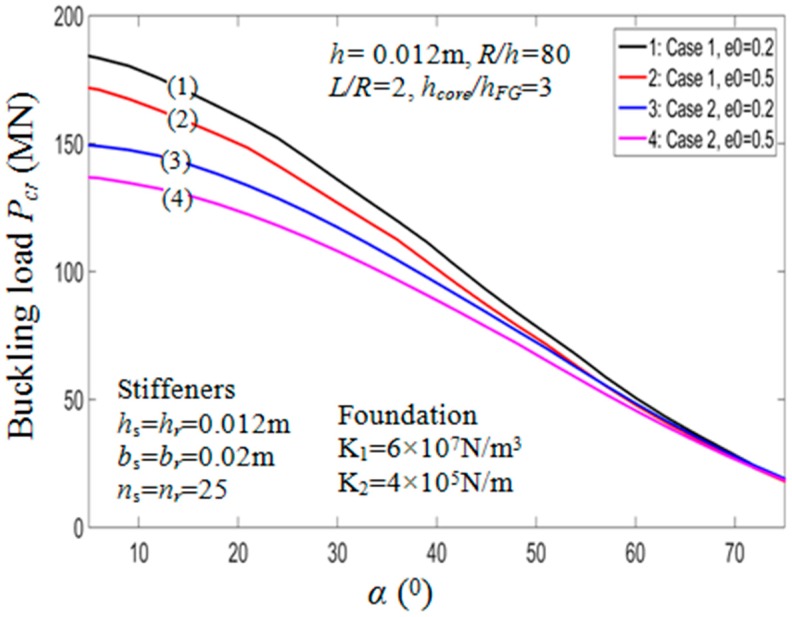
Effects of semi-vertex angle *α* on critical load *P_cr_* (*k*_2_ = *k*_3_ = *k* = 1). Case 1: Outside stiffener; Case 2: Inside stiffener.

**Figure 7:**
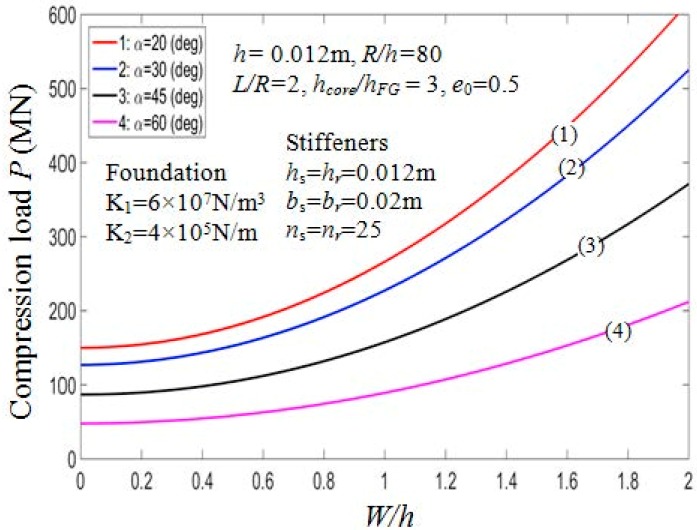
Effects of semi-vertex angle *α* on postbuckling load—deflection curves (Outside stiffener, *k*_2_ = *k*_3_ = *k* = 1).

The buckling loads of the PSTC in relation with the semi-vertex angle α are presented in Table 6. It could be noted from the table that when α increases, the critical buckling load of the PSTC decreases remarkably. Indeed, with $e_{0}=0.5$ in case 1, the value of $P_{cr}$ experiences a reduction from 171.8857 MN to 10.9997 MN (93.6%) when the value varies from 50° to 80°. This observation has also been mentioned in Ref. [11,18]. The variation of critical axial compressive loads in relation with the semi-vertex angle is plotted in Figure 6 for various porosity coefficients and both cases of stiffener arrangements. Also, the influence of angle α on the equilibrium behaviors of the PSTC with outer stiffeners in the post-buckling phase is presented in Figure 7. The figure also shows that, when the value of angle α increases, $P_{cr}$ decreases.

#### 7.2.3. Effect of Geometrical Ratios

**Figure 8:**
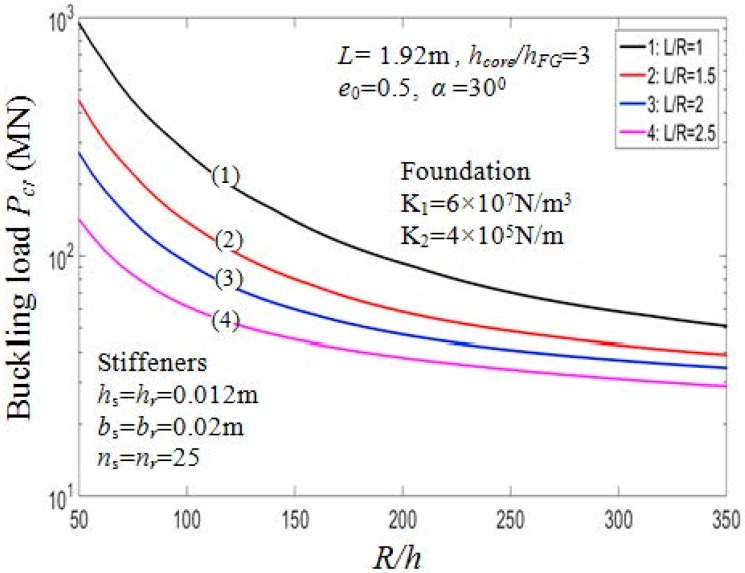
Effects of *R*/*h* and *L*/*R* on critical load *P_cr_* (Case 1, *k*_2_ = *k*_3_ = *k* = 1).

**Figure 9:**
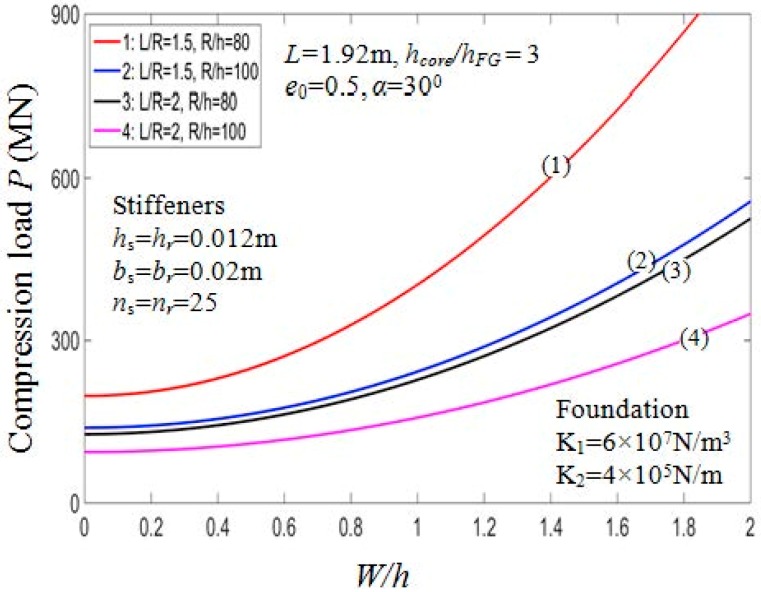
Effects of *R*/*h* and *L*/*R* on postbuckling load—deflection curves (Case 1, *k*_2_ = *k*_3_ = *k* = 1).

Effects of geometrical ratios L/R and R/h, on the buckling load $P_{cr}$ of the PSTC are presented in Table 7 and graphically illustrated in Figure 8. When L/R and R/h ratios increase, $P_{cr}$ decreases significantly. It is clear from the actual mechanical behavior of the structure that, in case of the shell structure, the thinner or the longer the shell, the smaller the value $P_{cr}$. Indeed, in Table 7, in the case of outside stiffeners, drawing the comparison between $P_{cr}=684.7950$ MN (when *R*/*h* = 60, *L*/*R* = 1) and $P_{cr}=197.9920$ MN (when *R*/*h* = 60, *L*/*R* = 2), the value of $P_{cr}$ decreases by approximately 71.1%. This trend is also depicted in Figure 9 for the effect of *R*/*h* and *L*/*R* ratios on the post-buckling equilibrium paths of the PSTC in the case 1. Thus, the bearing capacity of the shell is quite sensitive to the variation of *L*/*R* and *R*/*h* ratios.

#### 7.2.4. Effects of Volume Fraction Index

**Figure 10:**
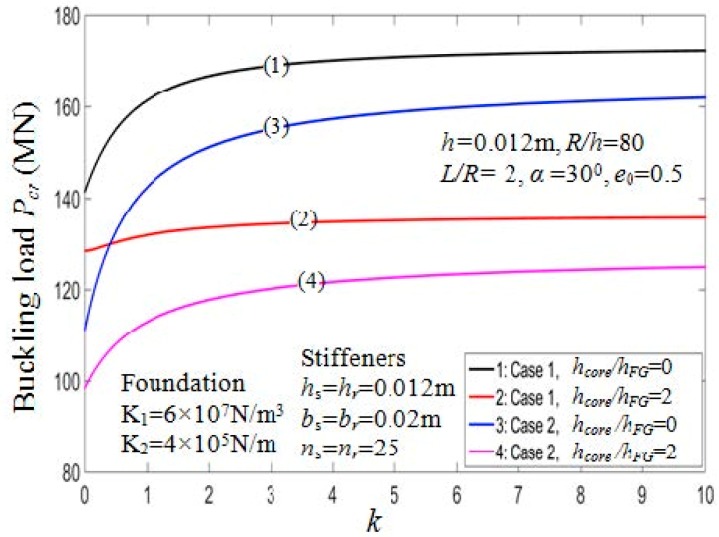
Effects of volume fraction indexes on critical load *P_cr_* (*k*_2_, *k*_3_ = 1/*k*).

**Figure 11:**
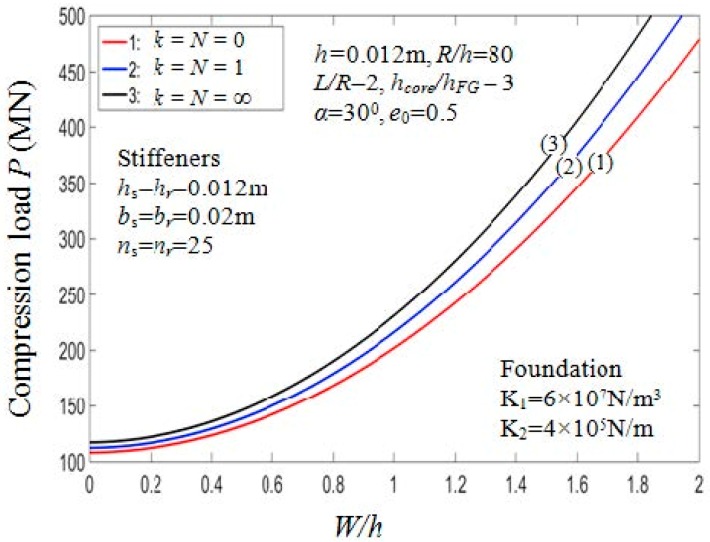
Effects of volume fraction indexes on postbuckling load—deflection curves (Case 1).

The critical buckling loads affected by the parameters k, $k_{2}$ and $k_{3}$ are shown in Table 8. The critical buckling loads vary according to the volume fraction index for two different values of the $h_{core}/h_{FG}$ ratio depicted in Figure 10. From the figure, when the value of *k* increases, the critical loads $P_{cr}$ increase. The reason is that the portion of the ceramic constituent in shell structure increase when the value of *k* increase. This is also confirmed by observing Figure 11, which depicts the load-deflection curves of the PSTC with outside stiffeners in relation to the volume fraction index in the post-buckling phase.

#### 7.2.5. Effect of Stiffeners and Foundation

The effects of stiffeners and elastic foundations on the buckling loads $P_{cr}$ of the PSTC are presented in Table 9. It is noted that the higher the number of stiffeners being used, the higher the buckling load. Indeed, for case 1 with $K_{1}=6\times 10^{7}$ N/m^3^, $K_{2}=4\times 10^{5}$ N/m, drawing the comparison between $P_{cr}=90.1237$ MN $\left(n_{s}=n_{r}=0\right)$ and $P_{cr}=161.2914$ MN $\left(n_{s}=n_{r}=50\right)$, we could recognize the increment in the value of critical compressive load by about 79%. Furthermore, the critical compressive loads $P_{cr}$ of the PSTC stiffened by rings are higher than that of the PSTC stiffened by stringers.

It is also noted that the presence of elastic foundations enhances the buckling loads. The buckling load of the PSTC increases according to the increment of the foundation parameters. Indeed, for the PSTC with orthogonal stiffeners with $\left(n_{s}=n_{r}=50\right)$ the value of $P_{cr}$ rises by about 9.3% from 119.4573MN with the absence of elastic foundation to 130.5622MN with the presence of elastic foundation: $K_{1}=9\times 10^{7}$ N/m^3^; $K_{2}=6\times 10^{5}$ N/m).

**Figure 12:**
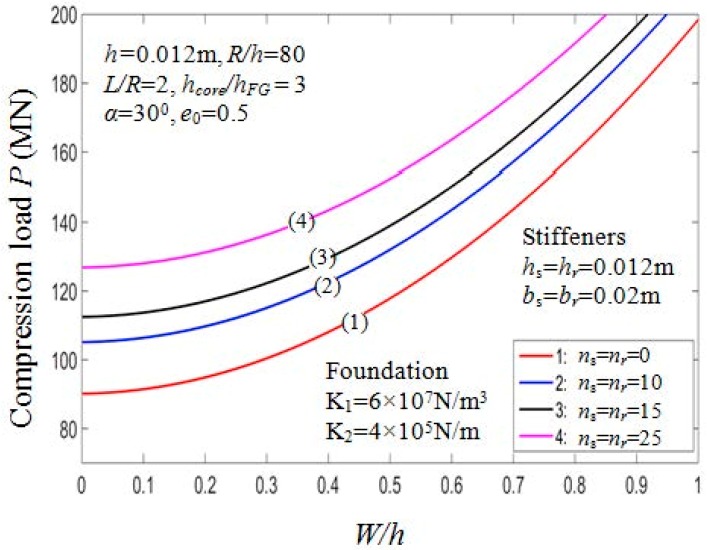
Effects of stiffeners on postbuckling load—deflection curves (Case 1, *k*_2_ = *k*_3_ = *k* = 1).

**Figure 13:**
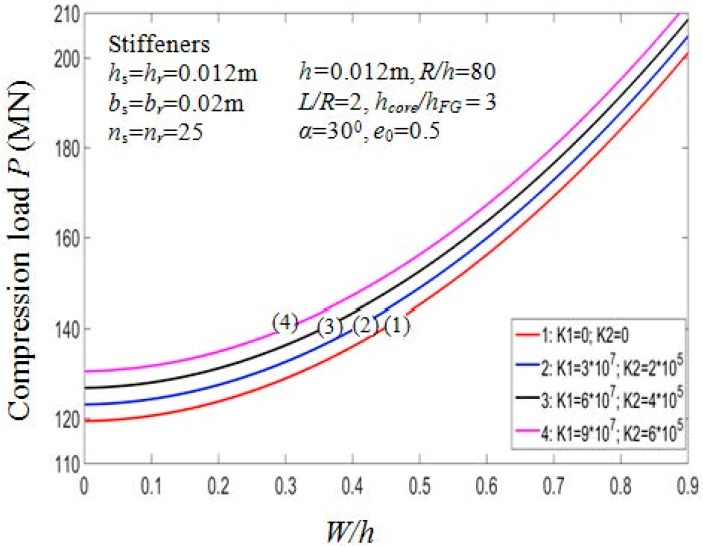
Effects of foundation on postbuckling load—deflection curves (Case 1, *k*_2_ = *k*_3_ = *k* = 1).

Figure 12 depicts the effect of stiffeners quantity on the post-buckling equilibrium path P−W/h of the PSTC. The value of the buckling loads is in a proportional relation with the quantity of the stiffeners. The curve for the stiffeners-free case and $n_{s}=n_{r}=25$ case bottoms and tops the graph, respectively. The curves for *n_s =_ n_r_* = 15 and *n_s_ = n_r_* = 10 locate in the middle range. The effect of foundation parameters on the post-buckling equilibrium paths P−W/h of the PSTC is also shown in Figure 13. It is observed that when the foundation parameters $K_{1},K_{2}$ increases, the curves gradually rise, in other words, the post-buckling equilibrium loads increase. From the figure, the curve for $K_{1}=9\times 10^{7}$ N/m^3^; $K_{2}=6\times 10^{5}$ N/m peaks, in other words, in this case, the buckling load at specific deflection value W/h is the highest among all the cases considered. The buckling load for the case with $K_{1}=6\times 10^{7}$ N/m^3^; $K_{2}=4\times 10^{5}$ N/m is greater than that for the case with $K_{1}=3\times 10^{7}$ N/m^3^; $K_{2}=2\times 10^{5}$ N/m in the post-buckling phase of the PSTC.

## 8. Conclusions

The paper produces an analytical procedure to analyze the nonlinear instability of the porous eccentrically stiffened functionally graded sandwich truncated conical shells surrounded by Pasternak elastic foundations using displacement approach. The core is made of a porous material (metal foam) with properties varying across its thickness according to a simple cosine law in term of a coefficient related to plate’s porosity. The material properties of FG coatings and stiffeners are assumed to be graded through the thickness direction according to a simple power law distribution in terms of the volume fractions of the constituents. Two cases of stiffener arrangement: outside and inside stiffened are considered. The smeared stiffeners technique with von Karman geometrical nonlinearity and the classical shell theory are employed to bring about the governing equations. The Galerkin method is employed to obtain theoretical expressions of load-deflection curves or the post-buckling equilibrium paths. The numerical results show that the reinforced stiffeners, with volume fraction index *k*, the length-to-radius ratio *L*/*R*, the radius-to-thickness ratio *R*/*h*, and foundation parameters $K_{1},K_{2}$ significantly influence the buckling and post-buckling
behaviors of the porous eccentrically stiffened functionally graded truncated conical sandwich shells. The study also shows the profound effects of the porosity coefficient $e_{0}$ and the core layer thickness on the critical buckling compressive loads and load-deflection curves in the post-buckling phase of the shell. Moreover, the stiffener arrangement has considerable influence on the critical buckling loads, the PSTC reinforced by outside stiffeners is always stiffer than that reinforced by inside stiffeners.

## Figures and Tables

**Table 1:** The geometric properties for the stiffened (un-stiffenedt) truncated conical shells.

Model	L/R	R/h	h (m)	α (^°^)	$h_{core}/h_{FG}\;$	$b_{r}=b_{s}\;(m)$	$h_{r}=h_{s}\;(m)$	$n_{r}\;$	$n_{s}\;$
M1	0.2; 0.5	100	0.01	1 to 80	-	-	-	-	-
M2	2	150	0.05	30	0 to 5	0.02	0.03	50	30
M3	2	150	0.01	45	0 to 8	-	-	-	-
M4	2	80	0.012	30	3	0.02	0.012	35	25

**Table 2:** Dimensionless buckling axial compressive loads of un-stiffened isotropic truncated conical shells without elastic foundation.

α	*L*/*R* = 0.2			*L*/*R* = 0.5		
	Naj et al. [2]	Baruch et al. [42]	Present $\left(P^{*}\right)$	Naj et al. [2]	Baruch et al. [42]	Present $\left(P^{*}\right)$
1°	1.005 (7)	1.005 (7)	1.0002 (1,12) ^a^	1.0017 (8)	1.002 (8)	1.0001 (2,17)
5°	1.006 (7)	1.006 (7)	1.0001 (1,12)	1.001 (8)	1.002 (8)	1.0002 (2,17)
10°	1.007 (7)	1.007 (7)	1.0002 (1,12)	1.000 (8)	1.002 (8)	1.0005 (2,17)
30°	1.0171 (5)	1.017 (5)	1.0017 (1,7)	0.987 (7)	1.001 (7)	1.0023 (2,15)
60°	1.148 (0)	1.144 (0)	1.1299 (1,1)	1.045 (7)	1.044 (7)	1.0150 (1,14)
80°	2.492 (0)	2.477 (0)	2.5091 (1,1)	1.004 (5)	1.015 (5)	1.0266 (1,4)

^a^ Buckling mode (*m*,*n*).

**Table 3:** Linear critical load of stiffened FG sandwich truncated conical shells.

$P_{cr}\;(\mathrm{MN})$	Case 1 (Outside Stiffeners)		Case 2 (Inside Stiffeners)	
*h_core_*/*h_FG_*	Dung et al. [21]	Present	Dung et al. [21]	Present
0	19.46667 (8,18)	19.4667 (8,18) ^a^	19.14549 (7,21)	19.1455 (7,21)
0.5	16.12768 (8,16)	16.1277 (8,16)	15.79773 (6,22)	15.7977 (6,22)
1	14.09267 (8,16)	14.0927 (8,16)	13.76594 (6,22)	13.7659 (6,22)
2	11.74586 (8,15)	11.7459 (8,15)	11.42875 (6,22)	11.4288 (6,22)
3	10.43697 (8,16)	10.4370 (8,16)	10.12653 (6,22)	10.1265 (6,22)
4	9.60325 (8,16)	9.6033 (8,16)	9.29804 (6,22)	9.2980 (6,22)
5	9.02635 (8,16)	9.0264 (8,16)	8.72504 (6,22)	8.7250 (6,22)

^a^ Buckling mode (*m*,*n*).

**Table 4:** Comparisons of nondimensional critical axial loads (calculated by Equation (27)) for un-stiffened three-layered FG/Metal/FG truncated conical shells with various ratio *h_core_*/*h_FG_*.

$P_{cr}\;(\mathrm{GN})$	*k* = 1			*k* = 2			*k = 5*		
	Deniz [43]	Present	Error	Deniz [43]	Present	Error	Deniz [43]	Present	Error
*h_core_*/*h_FG_* = 0	1.244	1.2914 (6,22) ^a^	3.7%	1.314	1.3605 (6.22)	3.4%	1.390	1.4392 (6,22)	3.4%
*h_core_*/*h_FG_* = 2	1.190	1.1459 (6,22)	−3.8%	1.246	1.2021 (6,22)	−3.7%	1.297	1.2649 (6,22)	−3.8%
*h_core_*/*h_FG_* = 4	1.135	1.0915 (6,22)	−3.8%	1.178	1.1321 (6,22)	−3.5%	1.217	1.1713 (6,22)	−3.9%
*h_core_*/*h_FG_* = 6	1.105	1.0654 (6,23)	−3.6%	1.139	1.1086 (6,22)	−2.7%	1.171	1.1307 (6,22)	−3.6%
*h_core_*/*h_FG_* = 8	1.085	1.0502 (6,23)	−3.2%	1.113	1.0887 (6,22)	−2.2%	1.140	1.0968 (6,22)	−3.9%

^a^ Buckling mode (*m*,*n*).

**Table 5:** The critical buckling load $P_{cr}$ of the PSTC for various ratios $h_{core}/h_{FG}$.

*P_cr_* (MN)	Case 1: Outside Stiffener			Case 2: Inside Stiffener		
	$e_{0}=0.2\;$	$e_{0}=0.5\;$	$e_{0}=0.8\;$	$e_{0}=0.2\;$	$e_{0}=0.5\;$	$e_{0}=0.8\;$
*h_core_*/*h_FG_* = 0	161.4554 (7,1)	161.4554 (7,1)	161.4554 (7,1)	142.5447 (5,16)	142.5447 (5,16)	142.5447 (5,16)
*h_core_*/*h_FG_* = 0.5	152.0344 (7,1)	148.6324 (7,1)	145.2239 (7,1)	133.1968 (5,15)	129.6503 (5,15)	126.1000 (5,18)
*h_core_*/*h_FG_* = 1	146.3406 (7,1)	140.9428 (7,1)	135.5258 (7,1)	127.5050 (5,15)	121.9165 (5,15)	116.3167 (5,15)
*h_core_*/*h_FG_* = 2	139.6989 (7,1)	131.9538 (7,1)	124.1623 (7,1)	120.9373 (5,15)	112.9854 (5,15)	105.0065 (5,15)
*h_core_*/*h_FG_* = 3	135.9469 (7,1)	126.8605 (7,1)	117.7094 (7,1)	117.3071 (5,15)	108.0130 (5,15)	98.6459 (5,15)
*h_core_*/*h_FG_* = 4	133.5555 (7,1)	123.5999 (7,1)	113.5562 (7,1)	114.9159 (5,15)	104.7698 (5,15)	94.5725 (5,15)
*h_core_*/*h_FG_* = 5	131.8897 (7,1)	121.3272 (7,1)	110.6618 (7,1)	113.2905 (5,15)	102.5464 (5,15)	91.7425 (5,15)
*h_core_*/*h_FG_* = 10	127.8946 (7,1)	115.8633 (7,1)	103.6858 (7,1)	109.4029 (5,15)	97.2183 (5,15)	84.9488 (5,15)
*h_core_*/*h_FG_* = 20	125.4750 (7,1)	112.5450 (7,1)	99.4363 (7,1)	107.0552 (5,15)	93.9934 (5,15)	80.8281 (5,15)

**Table 6:** Critical compression load $P_{cr}$ for various semi-vertex angles α.

$P_{cr}$ (MN)	Case 1: Outside Stiffener		Case 2: Inside Stiffener	
	$e_{0}=0.2\;$	$e_{0}=0.5\;$	$e_{0}=0.2\;$	$e_{0}=0.5\;$
*α* = 5°	184.2470 (9,1)	171.8857 (9,1)	149.3844 (6,14)	136.8875 (6,14)
*α* = 10°	178.8700 (8,5)	166.0860 (8,3)	146.9110 (6,14)	133.9463 (5,14)
*α* = 20°	160.5859 (8,1)	150.1258 (8,1)	135.1141 (5,15)	123.7183 (5,15)
*α* = 30°	135.9469 (7,1)	126.8605 (7,1)	117.3071 (5,15)	108.0130 (5,15)
*α* = 45°	92.8172 (6,1)	86.9674 (6,1)	84.0426 (5,14)	78.3735 (5,14)
*α* = 60°	50.6289 (5,1)	47.8436 (5,1)	48.4738 (4,13)	45.6781 (4,12)
*α* = 70°	28.1649 (4,1)	26.8487 (4,1)	27.7523 (4,10)	26.5536 (4,9)
*α* = 80°	11.2994 (4,1)	10.9997 (4,1)	11.6098 (4,1)	11.3076 (4,2)

**Table 7:** Critical compression load $P_{cr}$ for various values of *L*/*R* and *R*/*h* ratios.

$P_{cr}$ (MN)	*R*/*h* = 60	*R*/*h* = 80	*R*/*h* = 100	*R*/*h* = 200	*R*/*h* = 300
**Case 1: Outside stiffeners**					
*L*/*R* = 1	684.7950 (3,9)	398.8262 (4,1)	272.3611 (5,1)	93.1743 (6,1)	58.5103 (7,1)
*L*/*R* = 1.5	320.8777 (5,1)	197.8373 (6,1)	139.0107 (6,1)	58.4647 (8,1)	42.7500 (9,1)
*L*/*R* = 2	197.9920 (6,1)	126.8605 (7,1)	94.1463 (8,1)	47.0167 (9,1)	37.0240 (9,7)
*L*/*R* = 3	109.9757 (8,1)	77.4973 (8,6)	61.4766 (9,1)	37.9004 (9,10)	30.9346 (8,13)
**Case 2: Inside stiffeners**					
*L*/*R* = 1	648.9722 (3,11)	379.1878 (3,14)	255.0781 (4,14)	82.7254 (5,20)	50.4788 (6,23)
*L*/*R* = 1.5	297.5119 (4,12)	177.0668 (4,15)	122.2705 (5,16)	47.5955 (6,20)	33.5999 (7,21)
*L*/*R* = 2	175.2790 (4,14)	108.0130 (5,15)	77.5981 (5,16)	35.7277 (6,18)	26.9945 (7,19)
*L*/*R* = 3	89.9204 (5,14)	60.0620 (6,15)	46.3523 (6,15)	26.2785 (7,16)	21.2033 (7,16)

**Table 8:** Critical compression load $P_{cr}$ for different values of volume fraction indexes.

$P_{cr}$ (MN)	Case 1: Outside Stiffener (*k*_2_ *= k*_3_ *=* 1/*k*)		Case 2: Inside Stiffener (*k*_2_ *= k*_3_ *= k*)	
	$e_{0}=0.2\;$	$e_{0}=0.5\;$	$e_{0}=0.2\;$	$e_{0}=0.5\;$
*k* = 0	135.4442 (7,1)	126.2111 (7,1)	105.4436 (5,16)	96.2521 (5,15)
*k* = 1	135.9469 (7,1)	126.8605 (7,1)	117.3071 (5,15)	108.0130 (5,15)
*k* = 5	137.5855 (7,1)	128.5525 (7,1)	125.4939 (5,15)	116.1792 (5,15)
*k* = 10	137.9374 (7,1)	128.9140 (7,1)	127.4802 (5,15)	118.1596 (5,15)
*k* = ∞	138.3158 (7,1)	129.3029 (7,1)	130.0065 (5,15)	120.6781 (5,15)

**Table 9:** Effects of stiffeners and foundation on buckling loads $P_{cr}$.

$P_{cr}$ (MN)	$K_{1}=0$ $K_{2}=0\;$	$K_{1}=3\times 10^{7}\;N/m^{3}$ $K_{2}=2\times 10^{5}\;N/m$	$K_{1}=6\times 10^{7}\;N/m^{3}$ $K_{2}=4\times 10^{5}\;N/m$	$K_{1}=9\times 10^{7}\;N/m^{3}$ $K_{2}=6\times 10^{5}\;N/m$
**Case 1: Conical Shell Reinforced by Outside Stiffener**				
*n_s_* = 0, *n_r_* = 0	81.8418 (5,16)	86.4019 (7,4)	90.1237 (7,4)	93.8390 (7,3)
*n_s_* = 50, *n_r_* = 0	96.7739 (2,16)	110.7194 (4,17)	118.5695 (5,14)	124.6769 (5,14)
*n_s_* = 0, *n_r_* = 50	111.2085 (8,1)	114.4478 (8,1)	117.6871 (8,1)	120.9264 (8,1)
*n_s_* = 25, *n_r_* = 25	119.4573 (7,1)	123.1589 (7,1)	126.8605 (7,1)	130.5622 (7,1)
*n_s_* = 50, *n_r_* = 50	153.6013 (6,9)	157.5907 (7,1)	161.2924 (7,1)	164.9940 (7,1)
**Case 2: Conical Shell Reinforced by Inside Stiffener**				
*n_s_* = 0, *n_r_* = 0	81.8418 (5,16)	86.4019 (7,4)	90.1237 (7,4)	93.8390 (7,3)
*n_s_* = 50, *n_r_* = 0	85.6480 (2,16)	101.4708 (3,17)	111.2717 (4,17)	120.2202 (4,17)
*n_s_* = 0, *n_r_* = 50	84.7967 (6,15)	89.6205 (6,15)	94.4443 (6,15)	99.2681 (6,15)
*n_s_* = 25, *n_r_* = 25	94.3881 (4,15)	101.8290 (5,15)	108.0130 (5,15)	114.1970 (5,15)
*n_s_* = 50, *n_r_* = 50	103.4412 (4,14)	112.0060 (4,14)	120.2306 (5,15)	126.4147 (5,15)

## Appendix A

$$\begin{array}{l} E_{1}=\int_{-h/2}^{h/2}E_{sh}dz=E_{c}h_{FG}+E_{mc}h_{FG}\frac{1}{k+1}+E_{m}\left[h_{core}-e_{0}\frac{2h_{core}}{\pi }\right]\\ E_{2}=\int_{-h/2}^{h/2}zE_{sh}dz=0\\ \begin{array}{ll} E_{3} & =\int_{-h/2}^{h/2}z^{2}E_{sh}dz=\frac{E_{c}}{12}\left[{\left(h_{FG}+h_{core}\right)}^{3}-h_{core}{}^{3}\right]\\ \; & +\frac{E_{mc}}{4}\left[\frac{h_{FG}^{3}}{k+3}-\frac{2h_{FG}^{2}\left(h_{FG}+h_{core}\right)}{k+2}+\frac{h_{FG}{\left(h_{FG}+h_{core}\right)}^{2}}{k+1}\right]+E_{m}\left[\frac{h_{core}{}^{3}}{12}-\frac{e_{0}h_{core}{}^{3}\left(\pi^{2}-8\right)}{2\pi^{3}}\right] \end{array} \end{array}$$

**Case 1:** Outside stiffener

$$\begin{array}{l} E_{1s}=E_{c}h_{s}+E_{mc}\frac{h_{s}}{k_{2}+1},\\ E_{2s}=E_{c}\frac{h_{s}^{2}+h_{s}(h_{FG}+h_{core})}{2}+E_{mc}\left(\frac{h_{s}^{2}}{k_{2}+2}+\frac{h_{s}(h_{FG}+h_{core})}{2k_{2}+2}\right),\\ E_{3s}=E_{c}\frac{3{\left(h_{Fg}+h_{core}\right)}^{2}h_{s}+6(h_{FG}+h_{core})h_{s}^{2}+4h_{s}^{3}}{12}+E_{mc}\left(\frac{h_{s}^{3}}{k_{2}+3}+\frac{h_{s}^{2}(h_{FG}+h_{core})}{k_{2}+2}+\frac{h_{s}{\left(h_{FG}+h_{core}\right)}^{2}}{4k_{2}+4}\right), \end{array}$$

$$\begin{array}{l} E_{1r}=E_{c}h_{r}+E_{mc}\frac{h_{r}}{k_{3}+1},\\ E_{2r}=E_{c}\frac{h_{r}^{2}+h_{r}(h_{FG}+h_{core})}{2}+E_{mc}\left(\frac{h_{r}^{2}}{k_{3}+2}+\frac{h_{r}(h_{FG}+h_{core})}{2k_{3}+2}\right),\\ E_{3r}=E_{c}\frac{3{\left(h_{Fg}+h_{core}\right)}^{2}h_{r}+6(h_{FG}+h_{core})h_{r}^{2}+4h_{r}^{3}}{12}+E_{mc}\left(\frac{h_{r}^{3}}{k_{3}+3}+\frac{h_{r}^{2}(h_{FG}+h_{core})}{k_{3}+2}+\frac{h_{r}{\left(h_{FG}+h_{core}\right)}^{2}}{4k_{3}+4}\right) \end{array}$$

**Case 2:** Inside stiffener

$$\begin{array}{l} E_{1s}=E_{c}h_{s}+E_{mc}\frac{h_{s}}{k_{2}+1},\\ E_{2s}=-E_{c}\frac{h_{s}(h_{FG}+h_{core})+h_{s}{}^{2}}{2}-E_{cm}\left(\frac{h_{s}{}^{2}}{k_{2}+2}+\frac{h_{s}(h_{FG}+h_{core})}{2k_{2}+2}\right),\\ E_{3s}=E_{c}\frac{3h_{s}{\left(h_{FG}+h_{core}\right)}^{2}+6h_{s}{}^{2}(h_{FG}+h_{core})+4h_{s}{}^{3}}{12}+E_{mc}\left(\frac{h_{s}{}^{3}}{k_{2}+3}+\frac{h_{s}{}^{2}(h_{FG}+h_{core})}{k_{2}+2}+\frac{h_{s}{\left(h_{FG}+h_{core}\right)}^{2}}{4k_{2}+4}\right)\; \end{array}$$

$$\begin{array}{l} E_{1r}=E_{c}h_{r}+E_{mc}\frac{h_{r}}{k_{3}+1},\\ E_{2r}=-E_{c}\frac{h_{r}(h_{FG}+h_{core})+h_{r}{}^{2}}{2}-E_{mc}\left(\frac{h_{r}{}^{2}}{k_{3}+2}+\frac{h_{r}(h_{FG}+h_{core})}{2k_{3}+2}\right),\\ E_{3r}=E_{c}\frac{3h_{r}{\left(h_{FG}+h_{core}\right)}^{2}+6h_{r}{}^{2}(h_{FG}+h_{core})+4h_{r}{}^{3}}{12}+E_{mc}\left(\frac{h_{r}{}^{3}}{k_{3}+3}+\frac{h_{r}{}^{2}(h_{FG}+h_{core})}{k_{3}+2}+\frac{h_{r}{\left(h_{FG}+h_{core}\right)}^{2}}{4k_{3}+4}\right)\; \end{array}$$

In Equation (9), $A_{11}=A_{22}=\frac{E_{1}}{1-\nu^{2}}$, $A_{12}=\nu A_{11}$; $A_{66}=\frac{1-\nu }{2}A_{11}$; $B_{11}=B_{22}=\frac{E_{2}}{1-\nu^{2}}$, $B_{12}=\nu B_{11}$; $B_{66}=\frac{1-\nu }{2}B_{11}$; $D_{11}=D_{22}=\frac{E_{3}}{1-\nu^{2}}$, $D_{12}=\nu D_{11}$; $D_{66}=\frac{1-\nu }{2}D_{11}$; $d_{1}\left(x\right)=\lambda_{0}x$, $d_{2}=\frac{L}{n_{r}}$, $e_{s}=\frac{h+h_{s}}{2}$, $e_{r}=\frac{h+h_{r}}{2}$, $C_{1}\left(x\right)=\frac{C_{1}^{0}}{x}$, $C_{1}^{0}=\frac{E_{2s}b_{s}}{\lambda_{0}}$, $C_{2}=\frac{E_{2r}b_{r}}{d_{2}}$, $\lambda_{0}=\frac{2\pi \sin\alpha }{n_{s}}$, in which $n_{s}$ is the number of stringers, $n_{r}$ is the number of rings; $b_{r}$ are the width of rings, $b_{s}$ is the width of stringers; $d_{1}=d_{1}\left(x\right)$ is the span between stringers; $d_{2}$ is the span between rings as shown in Figure 2; $e_{s}$ is the eccentricities of the stringers, $e_{r}$ is the eccentricities of the rings to the mid-surface of the shell as shown in Figure 1.

## Appendix B

In Equations (15)–(17)

$$\begin{array}{l} E_{1}=\int_{-h/2}^{h/2}E_{sh}dz=E_{c}h_{FG}+E_{mc}h_{FG}\frac{1}{k+1}+E_{m}\left[h_{core}-e_{0}\frac{2h_{core}}{\pi }\right]\\ E_{2}=\int_{-h/2}^{h/2}zE_{sh}dz=0\\ \begin{array}{ll} E_{3} & =\int_{-h/2}^{h/2}z^{2}E_{sh}dz=\frac{E_{c}}{12}\left[{\left(h_{FG}+h_{core}\right)}^{3}-h_{core}{}^{3}\right]\\ \; & +\frac{E_{mc}}{4}\left[\frac{h_{FG}^{3}}{k+3}-\frac{2h_{FG}^{2}\left(h_{FG}+h_{core}\right)}{k+2}+\frac{h_{FG}{\left(h_{FG}+h_{core}\right)}^{2}}{k+1}\right]+E_{m}\left[\frac{h_{core}{}^{3}}{12}-\frac{e_{0}h_{core}{}^{3}\left(\pi^{2}-8\right)}{2\pi^{3}}\right] \end{array} \end{array}$$

**Case 1:** Outside stiffener

$$\begin{array}{l} E_{1s}=E_{c}h_{s}+E_{mc}\frac{h_{s}}{k_{2}+1},\\ E_{2s}=E_{c}\frac{h_{s}^{2}+h_{s}(h_{FG}+h_{core})}{2}+E_{mc}\left(\frac{h_{s}^{2}}{k_{2}+2}+\frac{h_{s}(h_{FG}+h_{core})}{2k_{2}+2}\right),\\ E_{3s}=E_{c}\frac{3{\left(h_{Fg}+h_{core}\right)}^{2}h_{s}+6(h_{FG}+h_{core})h_{s}^{2}+4h_{s}^{3}}{12}+E_{mc}\left(\frac{h_{s}^{3}}{k_{2}+3}+\frac{h_{s}^{2}(h_{FG}+h_{core})}{k_{2}+2}+\frac{h_{s}{\left(h_{FG}+h_{core}\right)}^{2}}{4k_{2}+4}\right),\; \end{array}$$

$$\begin{array}{l} E_{1r}=E_{c}h_{r}+E_{mc}\frac{h_{r}}{k_{3}+1},\\ E_{2r}=E_{c}\frac{h_{r}^{2}+h_{r}(h_{FG}+h_{core})}{2}+E_{mc}\left(\frac{h_{r}^{2}}{k_{3}+2}+\frac{h_{r}(h_{FG}+h_{core})}{2k_{3}+2}\right),\\ E_{3r}=E_{c}\frac{3{\left(h_{Fg}+h_{core}\right)}^{2}h_{r}+6(h_{FG}+h_{core})h_{r}^{2}+4h_{r}^{3}}{12}+E_{mc}\left(\frac{h_{r}^{3}}{k_{3}+3}+\frac{h_{r}^{2}(h_{FG}+h_{core})}{k_{3}+2}+\frac{h_{r}{\left(h_{FG}+h_{core}\right)}^{2}}{4k_{3}+4}\right).\; \end{array}$$

**Case 2:** Inside stiffener

$$\begin{array}{l} E_{1s}=E_{c}h_{s}+E_{mc}\frac{h_{s}}{k_{2}+1},\\ E_{2s}=-E_{c}\frac{h_{s}(h_{FG}+h_{core})+h_{s}{}^{2}}{2}-E_{cm}\left(\frac{h_{s}{}^{2}}{k_{2}+2}+\frac{h_{s}(h_{FG}+h_{core})}{2k_{2}+2}\right),\\ E_{3s}=E_{c}\frac{3h_{s}{\left(h_{FG}+h_{core}\right)}^{2}+6h_{s}{}^{2}(h_{FG}+h_{core})+4h_{s}{}^{3}}{12}+E_{mc}\left(\frac{h_{s}{}^{3}}{k_{2}+3}+\frac{h_{s}{}^{2}(h_{FG}+h_{core})}{k_{2}+2}+\frac{h_{s}{\left(h_{FG}+h_{core}\right)}^{2}}{4k_{2}+4}\right)\; \end{array}$$

$$\begin{array}{l} E_{1r}=E_{c}h_{r}+E_{mc}\frac{h_{r}}{k_{3}+1},\\ E_{2r}=-E_{c}\frac{h_{r}(h_{FG}+h_{core})+h_{r}{}^{2}}{2}-E_{mc}\left(\frac{h_{r}{}^{2}}{k_{3}+2}+\frac{h_{r}(h_{FG}+h_{core})}{2k_{3}+2}\right),\\ E_{3r}=E_{c}\frac{3h_{r}{\left(h_{FG}+h_{core}\right)}^{2}+6h_{r}{}^{2}(h_{FG}+h_{core})+4h_{r}{}^{3}}{12}+E_{mc}\left(\frac{h_{r}{}^{3}}{k_{3}+3}+\frac{h_{r}{}^{2}(h_{FG}+h_{core})}{k_{3}+2}+\frac{h_{r}{\left(h_{FG}+h_{core}\right)}^{2}}{4k_{3}+4}\right)\; \end{array}$$

In Equation (9), $A_{11}=A_{22}=\frac{E_{1}}{1-\nu^{2}}$, $A_{12}=\nu A_{11}$; $A_{66}=\frac{1-\nu }{2}A_{11}$; $B_{11}=B_{22}=\frac{E_{2}}{1-\nu^{2}}$, $B_{12}=\nu B_{11}$; $B_{66}=\frac{1-\nu }{2}B_{11}$; $D_{11}=D_{22}=\frac{E_{3}}{1-\nu^{2}}$, $D_{12}=\nu D_{11}$; $D_{66}=\frac{1-\nu }{2}D_{11}$; $d_{1}\left(x\right)=\lambda_{0}x$, $d_{2}=\frac{L}{n_{r}}$, $e_{s}=\frac{h+h_{s}}{2}$, $e_{r}=\frac{h+h_{r}}{2}$, $C_{1}\left(x\right)=\frac{C_{1}^{0}}{x}$, $C_{1}^{0}=\frac{E_{2s}b_{s}}{\lambda_{0}}$, $C_{2}=\frac{E_{2r}b_{r}}{d_{2}}$, $\lambda_{0}=\frac{2\pi \sin\alpha }{n_{s}}$, in which $n_{s}$ is the number of stringers, $n_{r}$ is the number of rings; $b_{r}$ are the width of rings, $b_{s}$ is the width of stringers; $d_{1}=d_{1}\left(x\right)$ is the span between stringers; $d_{2}$ is the span between rings as shown in Figure 2; $e_{s}$ is the eccentricities of the stringers, $e_{r}$ is the eccentricities of the rings to the mid-surface of the shell as shown in Figure 1.

In Equations (15)–(17)

$$F_{11}=\left[xA_{11}+\frac{E_{1s}b_{s}}{\lambda_{o}}\right]\frac{\partial^{2}}{\partial x^{2}}+A_{66}\frac{1}{x\sin^{2}\alpha }\frac{\partial^{2}}{\partial \theta^{2}}+A_{11}\frac{\partial }{\partial x}-\left[A_{22}+\frac{E_{1r}b_{r}}{d_{2}}\right]\frac{1}{x}\;$$

$$F_{12}=\frac{1}{\sin\alpha }\left(A_{12}+A_{66}\right)\frac{\partial^{2}}{\partial x\partial \theta }-\left[A_{22}+A_{66}+\frac{E_{1r}b_{r}}{d_{2}}\right]\frac{1}{x\sin\alpha }\frac{\partial }{\partial \theta }\;$$

$$\begin{array}{ll} F_{13}=- & \left[xB_{11}+C_{1}^{o}\right]\frac{\partial^{3}}{\partial x^{3}}-\frac{1}{x\sin^{2}\alpha }\left[B_{12}+2B_{66}\right]\frac{\partial^{3}}{\partial x\partial \theta^{2}}-B_{11}\frac{\partial^{2}}{\partial x^{2}}\\ \; & +\frac{1}{x^{2}\sin^{2}\alpha }\left(B_{12}+2B_{66}+B_{22}+C_{2}\right)\frac{\partial^{2}}{\partial \theta^{2}}+\left[A_{12}\cot\alpha +\frac{1}{x}\left(B_{22}+C_{2}\right)\right]\frac{\partial }{\partial x}-\frac{1}{x}\left(A_{22}+\frac{E_{1r}b_{r}}{d_{2}}\right)\cot\alpha \; \end{array}$$

$$F_{21}=\frac{1}{\sin\alpha }\left[A_{12}+A_{66}\right]\frac{\partial^{2}}{\partial x\partial \theta }+\frac{1}{x\sin\alpha }\left[A_{22}+A_{66}+\frac{E_{1r}b_{r}}{d_{2}}\right]\frac{\partial }{\partial \theta }\;$$

$$F_{22}=\left[A_{22}+\frac{E_{1r}b_{r}}{d_{2}}\right]\frac{1}{x\sin^{2}\alpha }\frac{\partial^{2}}{\partial \theta^{2}}+xA_{66}\frac{\partial^{2}}{\partial x^{2}}+A_{66}\frac{\partial }{\partial x}-A_{66}\frac{1}{x}\;$$

$$\begin{array}{l} F_{23}=-\frac{1}{\sin\alpha }\left[B_{12}+2B_{66}\right]\frac{\partial^{3}}{\partial x^{2}\partial \theta }-\frac{1}{x^{2}\sin^{3}\alpha }\left[B_{22}+C_{2}\right]\frac{\partial^{3}}{\partial \theta^{3}}-\frac{1}{x\sin\alpha }\left[B_{22}+C_{2}\right]\frac{\partial^{2}}{\partial x\partial \theta }\\ +\left[A_{22}+\frac{E_{1r}b_{r}}{d_{2}}\right]\frac{1}{x\sin\alpha }\frac{\partial }{\partial \theta }\cot\alpha \; \end{array}$$

$$\begin{array}{l} F_{31}=\left[xB_{11}+C_{1}^{o}\right]\frac{\partial^{3}}{\partial x^{3}}+\frac{1}{x\sin^{2}\alpha }\left[B_{12}+2B_{66}\right]\frac{\partial^{3}}{\partial x\partial \theta^{2}}+2B_{11}\frac{\partial^{2}}{\partial x^{2}}+\frac{1}{x^{2}\sin^{2}\alpha }\left[B_{22}+C_{2}\right]\frac{\partial^{2}}{\partial \theta^{2}}\\ -\left[\frac{1}{x}\left[B_{22}+C_{2}\right]+A_{12}\cot\alpha \right]\frac{\partial }{\partial x}+\left[B_{22}+C_{2}\right]\frac{1}{x^{2}}-\left[A_{22}+\frac{E_{1r}b_{r}}{d_{2}}\right]\frac{1}{x}\cot\alpha \; \end{array}$$

$$\begin{array}{l} F_{32}=\frac{1}{\sin\alpha }\left[B_{12}+2B_{66}\right]\frac{\partial^{3}}{\partial x^{2}\partial \theta }+\frac{1}{x^{2}\sin^{3}\alpha }\left[B_{22}+C_{2}\right]\frac{\partial^{3}}{\partial \theta^{3}}-\left[B_{22}+C_{2}\right]\frac{1}{x\sin\alpha }\frac{\partial^{2}}{\partial x\partial \theta }+\\ +\left[\left(B_{22}+C_{2}\right)\frac{1}{x^{2}\sin\alpha }-\left(A_{22}+\frac{E_{1r}b_{r}}{d_{2}}\right)\frac{1}{x\sin\alpha }\cot\alpha \right]\frac{\partial }{\partial \theta }\; \end{array}$$

$$\begin{array}{ll} F_{33}= & -\left[xD_{11}+\frac{E_{3s}b_{s}}{\lambda_{o}}\right]\frac{\partial^{4}}{\partial x^{4}}-\left[D_{22}+\frac{E_{3r}b_{r}}{d_{2}}\right]\frac{1}{x^{3}\sin^{4}\alpha }\frac{\partial^{4}}{\partial \theta^{4}}-\frac{2}{x\sin^{2}\alpha }\left[D_{12}+2D_{66}\right]\frac{\partial^{4}}{\partial x^{2}\partial \theta^{2}}\\ \; & +\frac{2}{x^{2}\sin^{2}\alpha }\left[D_{12}+2D_{66}\right]\frac{\partial^{3}}{\partial x\partial \theta^{2}}-2D_{11}\frac{\partial^{3}}{\partial x^{3}}+\left[\frac{1}{x}\left(D_{22}+\frac{E_{3r}b_{r}}{d_{2}}\right)+2B_{12}\cot\alpha \right]\frac{\partial^{2}}{\partial x^{2}}\\ \; & +\frac{2}{x^{2}\sin^{2}\alpha }\cot\alpha \left(B_{22}+C_{2}\right)\frac{\partial^{2}}{\partial \theta^{2}}-\frac{2}{x^{3}\sin^{2}\alpha }\left[D_{12}+2D_{66}+D_{22}+\frac{E_{3r}b_{r}}{d_{2}}\right]\frac{\partial^{2}}{\partial \theta^{2}}\\ \; & -\frac{1}{x^{2}}\left[D_{22}+\frac{E_{3r}b_{r}}{d_{2}}\right]\frac{\partial }{\partial x}+\left[B_{22}+C_{2}\right]\frac{1}{x^{2}}\cot\alpha -\frac{1}{x}\left[A_{22}+\frac{E_{1r}b_{r}}{d_{2}}\right]\cot^{2}\alpha \\ \; & -xK_{1}+xK_{2}\left(\frac{\partial^{2}}{\partial x^{2}}+\frac{1}{x}\frac{\partial }{\partial x}+\frac{1}{x^{2}\sin^{2}\alpha }\frac{\partial^{2}}{\partial \theta^{2}}\right)\; \end{array}$$

$$F_{34}=-\frac{P}{\pi \sin2\alpha }\frac{\partial^{2}}{\partial x^{2}}\;$$

$$\begin{array}{l} G_{14}=\left[xA_{11}+\frac{E_{1s}b_{s}}{\lambda_{o}}\right]\frac{\partial w}{\partial x}\frac{\partial^{2}w}{\partial x^{2}}+\frac{1}{2}\left[A_{11}-A_{12}\right]{\left(\frac{\partial w}{\partial x}\right)}^{2}+\frac{A_{12}}{2x\sin^{2}\alpha }\frac{\partial }{\partial x}{\left(\frac{\partial w}{\partial \theta }\right)}^{2}\\ -\frac{1}{2x^{2}\sin^{2}\alpha }\left(A_{12}+A_{22}+\frac{E_{1r}b_{r}}{d_{2}}\right){\left(\frac{\partial w}{\partial \theta }\right)}^{2}+\frac{1}{x\sin^{2}\alpha }A_{66}\frac{\partial^{2}w}{\partial x\partial \theta }\frac{\partial w}{\partial \theta }+\frac{1}{x\sin^{2}\alpha }A_{66}\frac{\partial w}{\partial x}\frac{\partial^{2}w}{\partial \theta^{2}}\; \end{array}$$

$$\begin{array}{l} G_{24}=\frac{1}{2\sin\alpha }A_{12}\frac{\partial }{\partial \theta }{\left(\frac{\partial w}{\partial x}\right)}^{2}+\frac{1}{x^{2}\sin^{3}\alpha }\left[A_{22}+\frac{E_{1r}b_{r}}{d_{2}}\right]\frac{\partial w}{\partial \theta }\frac{\partial^{2}w}{\partial \theta^{2}}+A_{66}\frac{1}{\sin\alpha }\frac{\partial^{2}w}{\partial x^{2}}\frac{\partial w}{\partial \theta }\\ +A_{66}\frac{1}{\sin\alpha }\frac{\partial w}{\partial x}\frac{\partial^{2}w}{\partial x\partial \theta }+A_{66}\frac{1}{x\sin\alpha }\frac{\partial w}{\partial x}\frac{\partial w}{\partial \theta }\; \end{array}$$

$$\begin{array}{cc} G_{34}= & B_{11}\frac{\partial w}{\partial x}\frac{\partial^{2}w}{\partial x^{2}}+\frac{1}{x^{3}\sin^{2}\alpha }B_{12}{\left(\frac{\partial w}{\partial \theta }\right)}^{2}-\frac{2}{x^{2}\sin^{2}\alpha }B_{12}\frac{\partial^{2}w}{\partial x\partial \theta }\frac{\partial w}{\partial \theta }+\frac{2}{x\sin^{2}\alpha }B_{12}{\left(\frac{\partial^{2}w}{\partial x\partial \theta }\right)}^{2}\\ \; & +2B_{66}\frac{1}{x\sin^{2}\alpha }\frac{\partial^{2}w}{\partial x^{2}}\frac{\partial^{2}w}{\partial \theta^{2}}-3B_{12}\frac{\partial w}{\partial x}\frac{\partial^{2}w}{\partial x^{2}}+\left[B_{22}+C_{2}\right]\frac{1}{x^{3}\sin^{2}\alpha }{\left(\frac{\partial w}{\partial \theta }\right)}^{2}\\ \; & -\left[B_{22}+C_{2}\right]\frac{2}{x^{2}\sin^{2}\alpha }\frac{\partial w}{\partial \theta }\frac{\partial^{2}w}{\partial x\partial \theta }+\frac{1}{2}A_{12}{\left(\frac{\partial w}{\partial x}\right)}^{2}\cot\alpha +\left[A_{22}+\frac{E_{1r}b_{r}}{d_{2}}\right]\frac{1}{2x^{2}\sin^{2}\alpha }{\left(\frac{\partial w}{\partial \theta }\right)}^{2}\cot\alpha \\ \; & +\left[xA_{11}+\frac{E_{1s}b_{s}}{\lambda_{o}}\right]\frac{\partial^{2}u}{\partial x^{2}}\frac{\partial w}{\partial x}+\left[xA_{11}+\frac{E_{1s}b_{s}}{\lambda_{o}}\right]\frac{\partial u}{\partial x}\frac{\partial^{2}w}{\partial x^{2}}+A_{11}\frac{\partial u}{\partial x}\frac{\partial w}{\partial x}+A_{12}\frac{\partial u}{\partial x}\frac{\partial w}{\partial x}+A_{12}u\frac{\partial^{2}w}{\partial x^{2}}\\ \; & +A_{12}\frac{1}{\sin\alpha }\frac{\partial^{2}v}{\partial x\partial \theta }\frac{\partial w}{\partial x}+A_{12}\frac{1}{\sin\alpha }\frac{\partial v}{\partial \theta }\frac{\partial^{2}w}{\partial x^{2}}+\frac{3}{2}\left[xA_{11}+\frac{E_{1s}b_{s}}{\lambda_{o}}\right]{\left(\frac{\partial w}{\partial x}\right)}^{2}\frac{\partial^{2}w}{\partial x^{2}}+\frac{1}{2}A_{11}{\left(\frac{\partial w}{\partial x}\right)}^{3}\\ \; & +A_{12}w\frac{\partial^{2}w}{\partial x^{2}}\cot\alpha -A_{12}\frac{1}{2x^{2}\sin^{2}\alpha }\frac{\partial w}{\partial x}{\left(\frac{\partial w}{\partial \theta }\right)}^{2}+A_{12}\frac{1}{2x\sin^{2}\alpha }\frac{\partial^{2}w}{\partial x^{2}}{\left(\frac{\partial w}{\partial \theta }\right)}^{2}+A_{12}\frac{2}{x\sin^{2}\alpha }\frac{\partial w}{\partial x}\frac{\partial w}{\partial \theta }\frac{\partial^{2}w}{\partial x\partial \theta }\\ \; & +B_{12}\frac{1}{x^{2}\sin^{2}\alpha }\frac{\partial w}{\partial x}\frac{\partial^{2}w}{\partial \theta^{2}}-B_{12}\frac{2}{x\sin^{2}\alpha }\frac{\partial^{2}w}{\partial x^{2}}\frac{\partial^{2}w}{\partial \theta^{2}}-A_{66}\frac{1}{x^{2}\sin^{2}\alpha }\frac{\partial u}{\partial \theta }\frac{\partial w}{\partial \theta }+A_{66}\frac{1}{x\sin^{2}\alpha }\frac{\partial^{2}u}{\partial x\partial \theta }\frac{\partial w}{\partial \theta }\\ \; & +A_{66}\frac{2}{x\sin^{2}\alpha }\frac{\partial u}{\partial \theta }\frac{\partial^{2}w}{\partial x\partial \theta }+A_{66}\frac{1}{\sin\alpha }\frac{\partial^{2}v}{\partial x^{2}}\frac{\partial w}{\partial \theta }+A_{66}\frac{2}{\sin\alpha }\frac{\partial v}{\partial x}\frac{\partial^{2}w}{\partial x\partial \theta }-A_{66}\frac{1}{x\sin\alpha }\frac{\partial v}{\partial x}\frac{\partial w}{\partial \theta }\\ \; & +A_{66}\frac{1}{x^{2}\sin\alpha }v\frac{\partial w}{\partial \theta }\\ \; & -A_{66}\frac{2}{x\sin\alpha }v\frac{\partial^{2}w}{\partial x\partial \theta }-A_{66}\frac{2}{x^{2}\sin^{2}\alpha }\frac{\partial w}{\partial \theta }\frac{\partial^{2}w}{\partial x\partial \theta }+A_{66}\frac{1}{x\sin^{2}\alpha }\frac{\partial^{2}w}{\partial x^{2}}{\left(\frac{\partial w}{\partial \theta }\right)}^{2}\\ \; & +A_{66}\frac{4}{x\sin^{2}\alpha }\frac{\partial w}{\partial x}\frac{\partial w}{\partial \theta }\frac{\partial^{2}w}{\partial x\partial \theta }+B_{66}\frac{8}{x^{2}\sin^{2}\alpha }\frac{\partial^{2}w}{\partial x\partial \theta }\frac{\partial w}{\partial \theta }-B_{66}\frac{4}{x^{3}\sin^{2}\alpha }{\left(\frac{\partial w}{\partial \theta }\right)}^{2}+A_{66}\frac{1}{x\sin^{2}\alpha }\frac{\partial^{2}u}{\partial \theta^{2}}\frac{\partial w}{\partial x}\\ \; & +A_{66}\frac{1}{\sin\alpha }\frac{\partial^{2}v}{\partial x\partial \theta }\frac{\partial w}{\partial x}-A_{66}\frac{1}{x\sin\alpha }\frac{\partial v}{\partial \theta }\frac{\partial w}{\partial x}+A_{66}\frac{1}{x\sin^{2}\alpha }{\left(\frac{\partial w}{\partial x}\right)}^{2}\frac{\partial^{2}w}{\partial \theta^{2}}-2B_{66}\frac{1}{x\sin^{2}\alpha }{\left(\frac{\partial^{2}w}{\partial x\partial \theta }\right)}^{2}\\ \; & +2B_{66}\frac{1}{x^{2}\sin^{2}\alpha }\frac{\partial^{2}w}{\partial \theta^{2}}\frac{\partial w}{\partial x}+A_{12}\frac{1}{x\sin^{2}\alpha }\frac{\partial^{2}u}{\partial x\partial \theta }\frac{\partial w}{\partial \theta }+A_{12}\frac{1}{x\sin^{2}\alpha }\frac{\partial u}{\partial x}\frac{\partial^{2}w}{\partial \theta^{2}}\\ \; & +\left[A_{22}+\frac{E_{1r}b_{r}}{d_{2}}\right]\frac{1}{x^{2}\sin^{2}\alpha }\frac{\partial u}{\partial \theta }\frac{\partial w}{\partial \theta }\\ \; & +\left[A_{22}+\frac{E_{1r}b_{r}}{d_{2}}\right]\frac{1}{x^{2}\sin^{2}\alpha }u\frac{\partial^{2}w}{\partial \theta^{2}}+\left[A_{22}+\frac{E_{1r}b_{r}}{d_{2}}\right]\frac{1}{x^{2}\sin^{3}\alpha }\frac{\partial^{2}v}{\partial \theta^{2}}\frac{\partial w}{\partial \theta }\\ \; & +\left[A_{22}+\frac{E_{1r}b_{r}}{d_{2}}\right]\frac{1}{x^{2}\sin^{3}\alpha }\frac{\partial v}{\partial \theta }\frac{\partial^{2}w}{\partial \theta^{2}}+\frac{1}{2}A_{12}\frac{1}{x\sin^{2}\alpha }{\left(\frac{\partial w}{\partial x}\right)}^{2}\frac{\partial^{2}w}{\partial \theta^{2}}+\left[A_{22}+\frac{E_{1r}b_{r}}{d_{2}}\right]\frac{1}{x^{2}\sin^{2}\alpha }w\frac{\partial^{2}w}{\partial \theta^{2}}\cot\alpha \\ \; & +\left[A_{22}+\frac{E_{1r}b_{r}}{d_{2}}\right]\frac{3}{2x^{3}\sin^{4}\alpha }{\left(\frac{\partial w}{\partial \theta }\right)}^{2}\frac{\partial^{2}w}{\partial \theta^{2}}\\ \; & -\left[B_{22}+C_{2}\right]\frac{1}{x^{2}\sin^{2}\alpha }\frac{\partial w}{\partial x}\frac{\partial^{2}w}{\partial \theta^{2}}\; \end{array}$$

## Appendix C

In Equations (22)–(24)

$$\begin{array}{l} H_{11}=-\left(J_{11}A_{11}+J_{12}\frac{E_{1s}b_{s}}{\lambda_{o}}\right)\frac{m^{2}\pi^{2}}{L^{2}}\sin\alpha -J_{13}A_{66}\frac{1}{\sin\alpha }\frac{n^{2}}{4}-J_{13}\left(A_{22}+\frac{E_{1r}b_{r}}{d_{2}}\right)\sin\alpha -J_{14}A_{11}\frac{m\pi }{L}\sin\alpha \\ H_{12}=-J_{12}\left(A_{12}+A_{66}\right)\frac{nm\pi }{2L}+J_{15}\left(A_{22}+A_{66}+\frac{E_{1r}b_{r}}{d_{2}}\right)\frac{n}{2}\\ H_{13}=\left(J_{11}B_{11}+J_{12}C_{1}^{o}\right)\frac{m^{3}\pi^{3}}{L^{3}}\sin\alpha +J_{13}\frac{1}{\sin\alpha }\left(B_{12}+2B_{66}\right)\frac{n^{2}m\pi }{4L}+J_{12}A_{12}\frac{m\pi }{L}\sin\alpha \cot\alpha \\ +J_{13}\left(B_{22}+C_{2}\right)\frac{m\pi }{L}\sin\alpha -J_{15}\left(A_{22}+\frac{E_{1r}b_{r}}{d_{2}}\right)\sin\alpha \cot\alpha +J_{14}B_{11}\frac{m^{2}\pi^{2}}{L^{2}}\sin\alpha \; \end{array}$$

$$L_{14}=-\left(J_{18}A_{11}+J_{19}\frac{E_{1s}b_{s}}{\lambda_{o}}\right)\frac{m^{3}\pi^{3}}{L^{3}}\sin\alpha +\left(J_{112}A_{12}+J_{112}A_{66}-J_{110}A_{66}\right)\frac{n^{2}m\pi }{4L\sin\alpha }+J_{111}\left(A_{11}-A_{12}\right)\frac{m^{2}\pi^{2}}{2L^{2}}\sin\alpha \;$$

$$\begin{array}{l} H_{21}=-J_{22}\left(A_{12}+A_{66}\right)\frac{nm\pi }{2L}+J_{26}\left(A_{22}+A_{66}+\frac{E_{1r}b_{r}}{d_{2}}\right)\frac{n}{2}\\ H_{22}=-J_{23}\left(A_{22}+\frac{E_{1r}b_{r}}{d_{2}}\right)\frac{1}{\sin\alpha }\frac{n^{2}}{4}-J_{21}A_{66}\frac{m^{2}\pi^{2}}{L^{2}}\sin\alpha -J_{23}A_{66}\sin\alpha +J_{25}A_{66}\frac{m\pi }{L}\sin\alpha \\ H_{23}=J_{23}\left(A_{22}+\frac{E_{1r}b_{r}}{d_{2}}\right)\frac{n}{2}\cot\alpha +J_{22}\left(B_{12}+2B_{66}\right)\frac{nm^{2}\pi^{2}}{2L^{2}}+\frac{J_{24}}{\sin^{2}\alpha }\left(B_{22}+C_{2}\right)\frac{n^{3}}{8}-J_{26}\left(B_{22}+C_{2}\right)\frac{nm\pi }{2L}\; \end{array}$$

$$L_{24}=J_{27}(A_{66}+A_{12})\frac{nm^{2}\pi^{2}}{2L^{2}}-\frac{J_{29}}{\sin^{2}\alpha }\left(A_{22}+\frac{E_{1r}b_{r}}{d_{2}}\right)\frac{n^{3}}{8}-J_{28}A_{66}\frac{nm^{2}\pi^{2}}{2L^{2}}+J_{210}A_{66}\frac{nm\pi }{2L}\;$$

$$\begin{array}{l} H_{31}=\left(J_{32}B_{11}+J_{33}C_{1}^{o}\right)\frac{m^{3}\pi^{3}}{L^{3}}\sin\alpha +\frac{J_{34}}{\sin\alpha }\left(B_{12}+2B_{66}\right)\frac{n^{2}m\pi }{4L}+J_{34}\left(B_{22}+C_{2}\right)\frac{m\pi }{L}\sin\alpha +J_{33}A_{12}\frac{m\pi }{L}\sin\alpha \cot\alpha \\ -2J_{38}B_{11}\frac{m^{2}\pi^{2}}{L^{2}}\sin\alpha -\frac{J_{310}}{\sin\alpha }\left(B_{22}+C_{2}\right)\frac{n^{2}}{4}+J_{310}\left(B_{22}+C_{2}\right)\sin\alpha -J_{39}\left(A_{22}+\frac{E_{1r}b_{r}}{d_{2}}\right)\sin\alpha \cot\alpha \; \end{array}$$

$$\begin{array}{l} H_{32}=J_{33}\left(B_{12}+2B_{66}\right)\frac{nm^{2}\pi^{2}}{2L^{2}}+\frac{J_{35}}{\sin^{2}\alpha }\left(B_{22}+C_{2}\right)\frac{n^{3}}{8}-J_{35}\left(B_{22}+C_{2}\right)\frac{n}{2}\\ +J_{34}\left(A_{22}+\frac{E_{1r}b_{r}}{d_{2}}\right)\frac{n}{2}\cot\alpha +J_{39}\left(B_{22}+C_{2}\right)\frac{nm\pi }{2L}\; \end{array}$$

$$\begin{array}{ll} H_{33}= & -\left(J_{32}D_{11}+J_{33}\frac{E_{3s}b_{s}}{\lambda_{o}}\right)\frac{m^{4}\pi^{4}}{L^{4}}\sin\alpha -J_{36}\left(D_{22}+\frac{E_{3r}b_{r}}{d_{2}}\right)\frac{1}{\sin^{3}\alpha }\frac{n^{4}}{16}-\frac{2J_{34}}{\sin\alpha }\left(D_{12}+2D_{66}\right)\frac{n^{2}m^{2}\pi^{2}}{4L^{2}}\\ \; & -J_{34}\left(D_{22}+\frac{E_{3r}b_{r}}{d_{2}}\right)\frac{m^{2}\pi^{2}}{L^{2}}\sin\alpha -2J_{33}B_{12}\frac{m^{2}\pi^{2}}{L^{2}}\sin\alpha \cot\alpha -\frac{2J_{35}}{\sin\alpha }\cot\alpha \left(B_{22}+C_{2}\right)\frac{n^{2}}{4}\\ \; & +\frac{2J_{36}}{\sin\alpha }\left(D_{12}+2D_{66}+D_{22}+\frac{E_{3r}b_{r}}{d_{2}}\right)\frac{n^{2}}{4}+J_{35}\left(B_{22}+C_{2}\right)\sin\alpha \cot\alpha -J_{34}\left(A_{22}+\frac{E_{1r}b_{r}}{d_{2}}\right)\sin\alpha \cot^{2}\alpha \\ \; & -J_{32}K_{1}\sin\alpha -K_{2}J_{32}\frac{m^{2}\pi^{2}}{L^{2}}\sin\alpha -K_{2}\frac{J_{34}}{\sin\alpha }\frac{n^{2}}{4}-\frac{2J_{310}}{\sin\alpha }\left(D_{12}+2D_{66}\right)\frac{n^{2}m\pi }{4L}\\ \; & \;+2J_{38}D_{11}\frac{m^{3}\pi^{3}}{L^{3}}\sin\alpha -J_{310}\left(D_{22}+\frac{E_{3r}b_{r}}{d_{2}}\right)\frac{m\pi }{L}\sin\alpha +J_{38}K_{2}\frac{m\pi }{L}\sin\alpha \; \end{array}$$

$$H_{34}=\frac{J_{33}m^{2}\pi }{2L^{2}\cos\alpha }\;$$

$$\begin{array}{ll} L_{34} & =J_{314}\frac{1}{\sin\alpha }B_{12}\frac{n^{2}}{4}+J_{314}\left(B_{22}+C_{2}\right)\frac{1}{\sin\alpha }\frac{n^{2}}{4}+J_{313}\left(A_{22}+\frac{E_{1r}b_{r}}{d_{2}}\right)\frac{1}{2\sin\alpha }\frac{n^{2}}{4}\cot\alpha -J_{314}B_{66}\frac{1}{\sin\alpha }n^{2}\\ \; & +\frac{1}{2}J_{316}A_{12}\frac{m^{2}\pi^{2}}{L^{2}}\sin\alpha \cot\alpha -J_{319}B_{11}\frac{m^{3}\pi^{3}}{L^{3}}\sin\alpha +3J_{319}B_{12}\frac{m^{3}\pi^{3}}{L^{3}}\sin\alpha -J_{320}B_{12}\frac{1}{\sin\alpha }\frac{n^{2}m\pi }{4L}\\ \; & -2J_{320}B_{66}\frac{1}{\sin\alpha }\frac{n^{2}m\pi }{4L}+J_{320}\left(B_{22}+C_{2}\right)\frac{1}{\sin\alpha }\frac{n^{2}m\pi }{4L}+2J_{322}B_{66}\frac{1}{\sin\alpha }\frac{n^{2}m^{2}\pi^{2}}{4L^{2}}-J_{322}A_{12}\frac{m^{2}\pi^{2}}{L^{2}}\sin\alpha \cot\alpha \\ \; & -J_{324}\left(A_{22}+\frac{E_{1r}b_{r}}{d_{2}}\right)\frac{\cot\alpha }{\sin\alpha }\frac{n^{2}}{4}-2J_{323}B_{12}\frac{1}{\sin\alpha }\frac{n^{2}m^{2}\pi^{2}}{4L^{2}}-\frac{J_{333}}{\sin\alpha }B_{12}\frac{n^{2}m\pi }{2L}-\left(B_{22}+C_{2}\right)\frac{J_{333}}{\sin\alpha }\frac{n^{2}m\pi }{2L}\\ \; & -J_{333}A_{66}\frac{1}{\sin\alpha }\frac{n^{2}m\pi }{2L}+2B_{66}\frac{J_{333}}{\sin\alpha }\frac{n^{2}m\pi }{L}+\frac{J_{336}}{\sin\alpha }B_{12}\frac{n^{2}m^{2}\pi^{2}}{2L^{2}}-\frac{J_{336}}{\sin\alpha }B_{66}\frac{n^{2}m^{2}\pi^{2}}{2L^{2}}\; \end{array}$$

$$\begin{array}{ll} L_{35} & =-A_{66}J_{311}\frac{nm^{2}\pi^{2}}{2L^{2}}+J_{313}A_{66}\frac{n}{2}-J_{313}\left(A_{22}+\frac{E_{1r}b_{r}}{d_{2}}\right)\frac{1}{\sin^{2}\alpha }\frac{n^{3}}{8}-J_{316}A_{12}\frac{nm^{2}\pi^{2}}{2L^{2}}-J_{316}A_{66}\frac{nm^{2}\pi^{2}}{2L^{2}}\\ \; & +J_{319}A_{66}\frac{nm\pi }{2L}+J_{322}A_{12}\frac{nm^{2}\pi^{2}}{2L^{2}}+J_{324}\left(A_{22}+\frac{E_{1r}b_{r}}{d_{2}}\right)\frac{1}{\sin^{2}\alpha }\frac{n^{3}}{8}-\frac{3}{2}J_{332}A_{66}\frac{nm\pi }{L}+J_{335}A_{66}\frac{nm^{2}\pi^{2}}{L^{2}}\; \end{array}$$

$$\begin{array}{ll} L_{36} & =-J_{312}A_{66}\frac{1}{\sin\alpha }\frac{n^{2}m\pi }{4L}-J_{312}A_{12}\frac{1}{\sin\alpha }\frac{n^{2}m\pi }{4L}-\left(J_{315}A_{11}+J_{316}\frac{E_{1s}b_{s}}{\lambda_{o}}\right)\frac{m^{3}\pi^{3}}{L^{3}}\sin\alpha \\ \; & -J_{317}A_{66}\frac{1}{\sin\alpha }\frac{n^{2}m\pi }{4L}-J_{319}A_{11}\frac{m^{2}\pi^{2}}{L^{2}}\sin\alpha -2J_{318}A_{12}\frac{m^{2}\pi^{2}}{L^{2}}\sin\alpha -J_{320}\left(A_{22}+\frac{E_{1r}b_{r}}{d_{2}}\right)\frac{1}{\sin\alpha }\frac{n^{2}}{4}\\ \; & +\left(J_{321}A_{11}+J_{322}\frac{E_{1s}b_{s}}{\lambda_{o}}\right)\frac{m^{3}\pi^{3}}{L^{3}}\sin\alpha +J_{323}A_{12}\frac{1}{\sin\alpha }\frac{n^{2}m\pi }{4L}-A_{66}\frac{J_{333}}{\sin\alpha }\frac{n^{2}}{4}+\frac{J_{333}}{\sin\alpha }\left(A_{22}+\frac{E_{1r}b_{r}}{d_{2}}\right)\frac{n^{2}}{4}\\ \; & +J_{336}A_{66}\frac{1}{\sin\alpha }\frac{n^{2}m\pi }{2L}\; \end{array}$$

$$\begin{array}{ll} L_{37}= & -\frac{3}{2}\left(J_{325}A_{11}+J_{326}\frac{E_{1s}b_{s}}{\lambda_{0}}\right)\frac{m^{4}\pi^{4}}{L^{4}}\sin\alpha -A_{66}\frac{J_{326}}{\sin\alpha }\frac{n^{2}m^{2}\pi^{2}}{4L^{2}}-A_{12}\frac{J_{327}}{2\sin\alpha }\frac{n^{2}m^{2}\pi^{2}}{4L^{2}}\\ \; & \;-A_{12}\frac{J_{328}}{2\sin\alpha }\frac{n^{2}m\pi }{4L}+\frac{1}{2}J_{329}A_{11}\frac{m^{3}\pi^{3}}{L^{3}}\sin\alpha -\frac{J_{330}}{\sin\alpha }A_{66}\frac{n^{2}m^{2}\pi^{2}}{4L}-\frac{J_{330}}{2\sin\alpha }A_{12}\frac{n^{2}m^{2}\pi^{2}}{4L^{2}}\\ \; & \;-J_{331}\left(A_{22}+\frac{E_{1r}b_{r}}{d_{2}}\right)\frac{3}{2\sin^{3}\alpha }\frac{n^{4}}{16}+A_{12}\frac{J_{334}}{\sin\alpha }\frac{n^{2}m^{3}\pi^{3}}{2L^{3}}+J_{334}A_{66}\frac{1}{\sin\alpha }\frac{n^{2}m^{3}\pi^{3}}{L^{3}}\; \end{array}$$

$$J_{11}=\pi \left[\begin{array}{l} \frac{{\left(L+x_{o}\right)}^{4}}{8}-\frac{x_{o}^{4}}{8}-\frac{3L^{2}x_{o}^{2}}{8\pi^{2}m^{2}}+\frac{3L^{2}{\left(L+x_{o}\right)}^{2}}{8\pi^{2}m^{2}} \end{array}\right]\;$$

$$J_{12}=\pi \left[\frac{{\left(L+x_{o}\right)}^{3}}{6}-\frac{x_{o}^{3}}{6}+\frac{L^{3}}{4\pi^{2}m^{2}}\right],\;$$

$$J_{13}=\pi \left(\frac{L^{2}}{4}+\frac{Lx_{o}}{2}\right)\;$$

$$J_{14}=-\frac{L^{2}\left(L+2x_{o}\right)}{4m},\;$$

$$J_{15}=-\frac{L^{2}}{4m}\;$$

$$J_{18}=\frac{4-4{\left(-1\right)}^{n}}{3n}\left[-\frac{L{\left(L+x_{o}\right)}^{3}{\left(-1\right)}^{m}}{3\pi m}+\frac{Lx_{o}^{3}}{3\pi m}-\frac{14L^{3}x_{o}}{9\pi^{3}m^{3}}+\frac{14L^{3}\left(L+x_{o}\right){\left(-1\right)}^{m}}{9\pi^{3}m^{3}}\right]\;$$

$$J_{19}=\frac{4-4{\left(-1\right)}^{n}}{3n}\left[-\frac{14L^{3}}{27\pi^{3}m^{3}}+\frac{Lx_{o}^{2}}{3\pi m}-\frac{L{\left(L+x_{o}\right)}^{2}{\left(-1\right)}^{m}}{3\pi m}+\frac{14L^{3}{\left(-1\right)}^{m}}{27\pi^{3}m^{3}}\right]\;$$

$$J_{110}=\frac{4-4{\left(-1\right)}^{n}}{3n}\left[-\frac{L\left(L+x_{o}\right){\left(-1\right)}^{m}}{3\pi m}+\frac{Lx_{o}}{3\pi m}\right]\;$$

$$J_{111}=-\frac{56{\left(-1\right)}^{n}-56}{27\pi^{2}m^{2}n}\left[L^{3}{\left(-1\right)}^{m}-L^{2}x_{o}+L^{2}x_{o}{\left(-1\right)}^{m}\right]\;$$

$$J_{112}=\frac{2}{9\pi mn}\left[L^{2}{\left(-1\right)}^{m+n}-L^{2}{\left(-1\right)}^{m}+Lx_{o}{\left(-1\right)}^{m+n}-x_{o}L{\left(-1\right)}^{m}-x_{o}L{\left(-1\right)}^{n}+x_{o}L\right]\;$$

$$J_{21}=\pi \left[\frac{{\left(L+x_{o}\right)}^{4}}{8}-\frac{x_{o}^{4}}{8}+\frac{3L^{2}x_{o}^{2}}{8\pi^{2}m^{2}}-\frac{3L^{2}{\left(L+x_{o}\right)}^{2}}{8\pi^{2}m^{2}}\right],\;$$

$$J_{22}=\pi \left[\begin{array}{l} \frac{{\left(L+x_{o}\right)}^{3}}{6}-\frac{x_{o}^{3}}{6}-\frac{L^{3}}{4\pi^{2}m^{2}} \end{array}\right]\;$$

$$J_{23}=\pi \left(\frac{L^{2}}{4}+\frac{Lx_{o}}{2}\right),\;$$

$$J_{24}=\frac{L\pi }{2},\;$$

$$J_{25}=-\frac{L^{2}\left(L+2x_{o}\right)}{4m},\;$$

$$J_{26}=-\frac{L^{2}}{4m}\;$$

$$J_{27}=\frac{1}{81\pi^{3}m^{3}n}[\begin{array}{l} 18\pi^{2}L^{3}m^{2}\cos^{3}\left(\pi m\right)\cos^{3}\left(\pi n\right)-18\pi^{2}L^{3}m^{2}\cos^{3}\left(\pi m\right)-4L^{3}\cos^{3}\left(\pi m\right)\cos^{3}\left(\pi n\right)\\ +4L^{3}\cos^{3}\left(\pi m\right)-72L^{3}\cos\left(\pi m\right)\cos\left(\pi n\right)+72L^{3}\cos\left(\pi m\right)\\ +48L^{3}\cos^{3}\left(\pi n\right)\sin^{2}\left(\frac{\pi m}{2}\right)+4L^{3}\cos^{3}\left(\pi n\right)-144L^{3}\cos\left(\pi n\right)\sin^{2}\left(\frac{\pi m}{2}\right)\\ +72L^{3}\cos\left(\pi n\right)+96L^{3}\sin^{2}\left(\frac{\pi m}{2}\right)-76L^{3}+36\pi^{2}L^{2}m^{2}x_{o}\cos^{3}\left(\pi m\right)\cos^{3}\left(\pi n\right)\\ -36\pi^{2}L^{2}m^{2}x_{o}\cos^{3}\left(\pi m\right)+18\pi^{2}Lm^{2}x_{o}^{2}\cos^{3}\left(\pi m\right)\cos^{3}\left(\pi n\right)\\ -18\pi^{2}Lm^{2}x_{o}^{2}\cos^{3}\left(\pi m\right)-18\pi^{2}Lm^{2}x_{o}^{2}\cos^{3}\left(\pi n\right)+18\pi^{2}Lm^{2}x_{o}^{2} \end{array}]$$

$$J_{28}=\frac{2-2{\left(-1\right)}^{n}}{3n}\left[-\frac{40L^{3}}{27\pi^{3}m^{3}}+\frac{2Lx_{o}{}^{2}}{3\pi m}-\frac{2L{\left(L+x_{o}\right)}^{2}{\left(-1\right)}^{m}}{3\pi m}+\frac{40L^{3}{\left(-1\right)}^{m}}{27\pi^{3}m^{3}}\right]\;$$

$$J_{29}=\frac{4L}{9\pi mn}\left[-{\left(-1\right)}^{n}+1+{\left(-1\right)}^{m+n}-{\left(-1\right)}^{m}\right]\;$$

$$J_{210}=\frac{4L^{2}}{27\pi^{2}m^{2}n}\left[{\left(-1\right)}^{m}-1+{\left(-1\right)}^{n}-{\left(-1\right)}^{m+n}\right]\;$$

$$J_{31}=\pi \left[\frac{{\left(L+x_{o}\right)}^{6}-x_{o}^{6}}{12}+\frac{5L^{2}\left(x_{o}^{4}-{\left(L+x_{o}\right)}^{4}\right)}{8\pi^{2}m^{2}}+\frac{15L^{4}\left({\left(L+x_{o}\right)}^{2}-x_{o}^{2}\right)}{8\pi^{4}m^{4}}\right]\;$$

$$J_{32}=\pi \left[\frac{{\left(L+x_{o}\right)}^{5}-x_{o}^{5}}{10}+\frac{3L^{5}}{4\pi^{4}m^{4}}+\frac{L^{2}\left(x_{o}^{3}-{\left(L+x_{o}\right)}^{3}\right)}{2\pi^{2}m^{2}}\right]\;$$

$$J_{33}=\pi \left[\frac{{\left(L+x_{o}\right)}^{4}-x_{o}^{4}}{8}-\frac{3L^{3}(L+2x_{o})}{8\pi^{2}m^{2}}\right],\;$$

$$J_{34}=J_{22},\;$$

$$J_{35}=J_{23},\;$$

$$J_{36}=J_{24}\;$$

$$J_{37}=\pi \left[\frac{L\left(x_{o}^{4}-{\left(L+x_{o}\right)}^{4}\right)}{4\pi m}+\frac{3L^{4}\left(L+2x_{o}\right)}{4\pi^{3}m^{3}}\right],\;$$

$$J_{38}=\pi \left[\frac{L\left(x_{o}^{3}-{\left(L+x_{o}\right)}^{3}\right)}{4\pi m}+\frac{3L^{4}}{8\pi^{3}m^{3}}\right]\;$$

$$J_{39}=\frac{-L^{2}\left(L+2x_{o}\right)}{4m},\;$$

$$J_{310}=-\frac{L^{2}}{4m}\;$$

$$J_{311}=\frac{2-2{\left(-1\right)}^{n}}{3n}\left[-\frac{2L{\left(L+x_{o}\right)}^{3}{\left(-1\right)}^{m}}{3\pi m}+\frac{40L^{3}\left(L+x_{o}\right){\left(-1\right)}^{m}}{9\pi^{3}m^{3}}+\frac{2Lx_{o}^{3}}{3m\pi }-\frac{40L^{3}x_{o}}{9\pi^{3}m^{3}}\right],\;$$

$$J_{312}=J_{28}\;$$

$$J_{313}=\frac{2-2{\left(-1\right)}^{n}}{3n}\left[\frac{2Lx_{o}}{3\pi m}-\frac{2L\left(L+x_{o}\right){\left(-1\right)}^{m}}{3\pi m}\right],\;$$

$$J_{314}=\frac{4L}{9\pi mn}\left[-{\left(-1\right)}^{n}+1+{\left(-1\right)}^{m+n}-{\left(-1\right)}^{m}\right]\;$$

$$J_{315}=\frac{4{\left(-1\right)}^{n}-4}{3n}\left\{\frac{L{\left(L+x_{o}\right)}^{4}{\left(-1\right)}^{m}-Lx_{o}^{4}}{3\pi m}+\frac{488L^{5}\left[{\left(-1\right)}^{m}-1\right]}{81\pi^{5}m^{5}}+\frac{28L^{3}x_{o}^{2}-28L^{3}{\left(L+x_{o}\right)}^{2}{\left(-1\right)}^{m}}{9\pi^{3}m^{3}}\right\}\;$$

$$J_{316}=\frac{4-4{\left(-1\right)}^{n}}{3n}\left[\begin{array}{l} -\frac{L{\left(L+x_{o}\right)}^{3}{\left(-1\right)}^{m}}{3\pi m}+\frac{14L^{3}\left(L+x_{o}\right){\left(-1\right)}^{m}}{9\pi^{3}m^{3}}+\frac{Lx_{o}^{3}}{3\pi m}-\frac{14L^{3}x_{o}}{9\pi^{3}m^{3}} \end{array}\right]\;$$

$$J_{317}=\frac{4-4{\left(-1\right)}^{n}}{3n}\left[-\frac{L{\left(L+x_{o}\right)}^{2}{\left(-1\right)}^{m}}{3\pi m}+\frac{14L^{3}{\left(-1\right)}^{m}}{27\pi^{3}m^{3}}+\frac{Lx_{o}^{2}}{3\pi m}-\frac{14L^{3}}{27\pi^{3}m^{3}}\right]\;$$

$$J_{318}=\frac{1-{\left(-1\right)}^{n}}{3n}\left[\frac{160L^{4}-160L^{4}{\left(-1\right)}^{m}}{27\pi^{4}m^{4}}+\frac{8L^{2}{\left(L+x_{o}\right)}^{2}{\left(-1\right)}^{m}}{3\pi^{2}m^{2}}-\frac{8L^{2}x_{o}^{2}}{3\pi^{2}m^{2}}\right]\;$$

$$J_{319}=\frac{1-{\left(-1\right)}^{n}}{3n}\left(\frac{16L^{2}\left(L+x_{o}\right){\left(-1\right)}^{m}}{9\pi^{2}m^{2}}-\frac{16L^{2}x_{o}}{9\pi^{2}m^{2}}\right)\;$$

$$J_{320}=\frac{8L^{2}}{27\pi^{2}m^{2}n}\left[-1+{\left(-1\right)}^{n}+{\left(-1\right)}^{m}-{\left(-1\right)}^{m+n}\right]\;$$

$$J_{321}=\frac{4-4{\left(-1\right)}^{n}}{3n}\left\{\begin{array}{l} \frac{2L\left[x_{o}^{4}-{\left(L+x_{o}\right)}^{4}{\left(-1\right)}^{m}\right]}{3\pi m}+\frac{1456L^{5}\left[1-{\left(-1\right)}^{m}\right]}{81\pi^{5}m^{5}}+\frac{80L^{3}\left[{\left(L+x_{o}\right)}^{2}{\left(-1\right)}^{m}-x_{o}^{2}\right]}{9\pi^{3}m^{3}} \end{array}\right\}\;$$

$$J_{322}=\frac{4-4{\left(-1\right)}^{n}}{3n}\left[-\frac{2L{\left(L+x_{o}\right)}^{3}{\left(-1\right)}^{m}}{3\pi m}+\frac{40L^{3}\left(L+x_{o}\right){\left(-1\right)}^{m}}{9\pi^{3}m^{3}}+\frac{2Lx_{o}^{3}}{3\pi m}-\frac{40L^{3}x_{o}}{9\pi^{3}m^{3}}\right]\;$$

$$J_{323}=\frac{4-4{\left(-1\right)}^{n}}{3n}\left[-\frac{2L{\left(L+x_{o}\right)}^{2}{\left(-1\right)}^{m}}{3\pi m}+\frac{40L^{3}{\left(-1\right)}^{m}}{27\pi^{3}m^{3}}+\frac{2Lx_{o}^{2}}{3\pi m}-\frac{40L^{3}}{27\pi^{3}m^{3}}\right]\;$$

$$J_{324}=\frac{4-4{\left(-1\right)}^{n}}{3n}\left[\frac{2Lx_{o}}{3\pi m}-\frac{2L\left(L+x_{o}\right){\left(-1\right)}^{m}}{3\pi m}\right]\;$$

$$J_{325}=-\frac{3\pi }{4}\left[\frac{x_{o}^{5}-{\left(L+x_{o}\right)}^{5}}{40}-\frac{3L^{5}}{256\pi^{4}m^{4}}+\frac{L^{2}\left({\left(L+x_{o}\right)}^{3}-x_{o}^{3}\right)}{32\pi^{2}m^{2}}\right]\;$$

$$J_{326}=-\frac{3\pi }{4}\left[\frac{{\left(L+x_{o}\right)}^{4}}{32}-\frac{x_{o}^{4}}{32}-\frac{3L^{3}(L+2x_{o})}{128\pi^{2}m^{2}}\right],\;$$

$$J_{327}=-\frac{3\pi }{4}\left[\frac{{\left(L+x_{o}\right)}^{3}}{24}-\frac{x_{o}^{3}}{24}-\frac{L^{3}}{64\pi^{2}m^{2}}\right]\;$$

$$J_{328}=\frac{\pi }{4}\left[\frac{3Lx_{o}}{32\pi m}-\frac{3L\left(L+x_{o}\right)}{32\pi m}\right],\;$$

$$J_{329}=-\frac{3\pi }{4}\left[\begin{array}{l} -\frac{51L^{4}}{256\pi^{3}m^{3}}+\frac{5L\left({\left(L+x_{o}\right)}^{3}-x_{o}^{3}\right)}{32\pi m} \end{array}\right]\;$$

$$J_{330}=\frac{\pi }{4}\left(\frac{{\left(L+x_{o}\right)}^{3}}{8}-\frac{x_{o}^{3}}{8}-\frac{15L^{3}}{64\pi^{2}m^{2}}\right),\;$$

$$J_{331}=\frac{3L\pi }{32\pi m}\;$$

$$J_{332}=\frac{16-16{\left(-1\right)}^{n}}{51\pi^{2}m^{2}n}\left[L^{3}{\left(-1\right)}^{m}-L^{2}x_{o}+L^{2}x_{o}{\left(-1\right)}^{m}\right]\;$$

$$J_{333}=\frac{4}{27\pi^{2}m^{2}n}\left[L^{2}{\left(-1\right)}^{m}-L^{2}+L^{2}{\left(-1\right)}^{n}-L^{2}{\left(-1\right)}^{m+n}\right],\;$$

$$J_{334}=\frac{\pi }{4}\left[\frac{{\left(L+x_{o}\right)}^{3}}{24}-\frac{x_{o}^{3}}{24}+\frac{L^{2}x_{o}}{64\pi^{2}m^{2}}\right]\;$$

$$J_{335}=\frac{-1}{81\pi^{3}m^{3}n}\left\{\begin{array}{l} 84L^{3}x_{o}\left[1-{\left(-1\right)}^{n}-{\left(-1\right)}^{m}+{\left(-1\right)}^{m+n}\right]+84L^{4}\left[{\left(-1\right)}^{m+n}-{\left(-1\right)}^{m}\right]\\ +18\pi^{3}m^{3}L^{4}{\left(-1\right)}^{m}+18\pi^{2}m^{2}Lx_{o}^{3}\left[-1-{\left(-1\right)}^{m+n}+{\left(-1\right)}^{m}+{\left(-1\right)}^{n}\right]\\ -18\pi^{2}m^{2}L^{4}{\left(-1\right)}^{m+n}+54\pi^{2}m^{2}\left[L^{2}x_{o}^{2}{\left(-1\right)}^{m}-L^{2}x_{o}^{2}{\left(-1\right)}^{m+n}\right]\\ +54\pi^{2}m^{2}\left[L^{3}x_{o}{\left(-1\right)}^{m}-L^{3}x_{o}{\left(-1\right)}^{m+n}\right] \end{array}\right\}\;$$

$$J_{336}=\frac{1}{81\pi^{3}m^{3}n}\left\{\begin{array}{l} 18\pi^{2}m^{2}L^{3}\left[{\left(-1\right)}^{m+n}-{\left(-1\right)}^{m}\right]+76L^{3}\left[-1-{\left(-1\right)}^{m+n}+{\left(-1\right)}^{m}+{\left(-1\right)}^{n}\right]\\ +18\pi^{2}m^{2}Lx_{o}^{2}\left[{\left(-1\right)}^{m+n}-{\left(-1\right)}^{m}-{\left(-1\right)}^{n}+1\right]\\ -96L^{3}{\left(-1\right)}^{n}\sin^{2}\left(\frac{\pi m}{2}\right)+96L^{3}\sin^{2}\left(\frac{\pi m}{2}\right)+36\pi^{2}m^{2}L^{2}x_{o}\left[{\left(-1\right)}^{m+n}-{\left(-1\right)}^{m}\right] \end{array}\right\}\;$$
